# Evolutionary Responses of a Reef-building Coral to Climate Change at the End of the Last Glacial Maximum

**DOI:** 10.1093/molbev/msac201

**Published:** 2022-10-11

**Authors:** Jia Zhang, Zoe T Richards, Arne A S Adam, Cheong Xin Chan, Chuya Shinzato, James Gilmour, Luke Thomas, Jan M Strugnell, David J Miller, Ira Cooke

**Affiliations:** Department of Molecular and Cell Biology, James Cook University, Townsville, QLD, 4811, Australia; Centre for Tropical Bioinformatics and Molecular Biology, James Cook University, Townsville, QLD, 4811, Australia; ARC Centre of Excellence for Coral Reef Studies, James Cook University, Townsville, QLD, 4811, Australia; Coral Conservation and Research Group, Trace and Environmental DNA Laboratory, School of Molecular and Life Sciences, Curtin University, Bentley, WA 6102, Australia; Collections and Research, Western Australian Museum, 49 Kew Street Welshpool, WA 6106, Australia; Coral Conservation and Research Group, Trace and Environmental DNA Laboratory, School of Molecular and Life Sciences, Curtin University, Bentley, WA 6102, Australia; The University of Queensland, School of Chemistry and Molecular Biosciences, Australian Centre for Ecogenomics, Brisbane, QLD 4072, Australia; Atmosphere and Ocean Research Institute, The University of Tokyo 277-8564, Chiba, Japan; Australia Institute of Marine Science, Indian Oceans Marine Research Centre, Crawley, WA, 6009, Australia; Australia Institute of Marine Science, Indian Oceans Marine Research Centre, Crawley, WA, 6009, Australia; Oceans Graduate School, The UWA Oceans Institute, The University of Western Australia, Perth, WA, 6009, Australia; Department of Marine Biology and Aquaculture, James Cook University, Townsville, QLD, 4811, Australia; Centre for Sustainable Fisheries and Aquaculture, James Cook University, Townsville, QLD, 4811, Australia; Department of Molecular and Cell Biology, James Cook University, Townsville, QLD, 4811, Australia; Centre for Tropical Bioinformatics and Molecular Biology, James Cook University, Townsville, QLD, 4811, Australia; ARC Centre of Excellence for Coral Reef Studies, James Cook University, Townsville, QLD, 4811, Australia; Marine Climate Change Unit, Okinawa Institute of Science and Technology, Onna-son, Okinawa, Japan 904-0495; Department of Molecular and Cell Biology, James Cook University, Townsville, QLD, 4811, Australia; Centre for Tropical Bioinformatics and Molecular Biology, James Cook University, Townsville, QLD, 4811, Australia

**Keywords:** *Acropora digitifera*, founder effects, glacial cycles, adaptive evolution, population genomics, selective sweeps

## Abstract

Climate change threatens the survival of coral reefs on a global scale, primarily through mass bleaching and mortality as a result of marine heatwaves. While these short-term effects are clear, predicting the fate of coral reefs over the coming century is a major challenge. One way to understand the longer-term effect of rapid climate change is to examine the response of coral populations to past climate shifts. Coastal and shallow-water marine ecosystems such as coral reefs have been reshaped many times by sea-level changes during the Pleistocene, yet few studies have directly linked this with its consequences on population demographics, dispersal, and adaptation. Here we use powerful analytical techniques, afforded by haplotype-phased whole-genomes, to establish such links for the reef-building coral, *Acropora digitifera*. We show that three genetically distinct populations are present in northwestern Australia, and that their rapid divergence since the last glacial maximum (LGM) can be explained by a combination of founder-effects and restricted gene flow. Signatures of selective sweeps, too strong to be explained by demographic history, are present in all three populations and overlap with genes that show different patterns of functional enrichment between inshore and offshore habitats. In contrast to rapid divergence in the host, we find that photosymbiont communities are largely undifferentiated between corals from all three locations, spanning almost 1000 km, indicating that selection on host genes, and not acquisition of novel symbionts, has been the primary driver of adaptation for this species in northwestern Australia.

## Introduction

Glacial cycling during the Pleistocene is thought to be a major driver of biodiversity dynamics (Hewitt 2000; Hofreiter and Stewart 2009), and its effects provide important lessons that can be used to help predict the impacts of future climate change (Hofreiter and Stewart 2009; Nogués-Bravo et al. 2018). Population genetics is a valuable tool to understand these past climate events because it can reveal historical changes in species’ demography, connectivity, and diversity. Widespread application of population genetic tools to terrestrial (Hofreiter and Stewart 2009) and marine (Mattingsdal et al. 2019) species in the northern hemisphere has revealed a predominant picture of persistence in southern refugia followed by expansion and northward migration after the last glacial maximum (LGM), with more recent work describing differential species’ responses depending on habitat requirements (Hofreiter and Stewart 2009) and patterns of dispersal (Mattingsdal et al. 2019). Much less is known about the impacts of past climate shifts on tropical marine systems such as coral reefs, despite the profound impacts that changes in temperature and sea level would have had on these shallow-water marine habitats (Wilson 2013; Ludt and Rocha 2015; Webster et al. 2018).

Throughout the tropics, the dominant effect of low sea levels during the LGM was a dramatic reduction in the amount of shallow water habitat (Kleypas 1997; Ludt et al. 2015). In broad agreement with this, many studies across a range of coral reef taxa have observed signatures of recent population expansion (Crandall et al. 2008; Crandall et al. 2012; Delrieu-Trottin et al. 2017); however, not all populations follow this pattern. Genome-wide approaches are now revealing differential demographic histories of cryptic and recently diverged populations (Bierne et al. 2003; Cooke et al. 2020; Underwood et al. 2020; Bongaerts et al. 2021), some of which show signatures of recent isolation and decline (Moran et al. 2019). Moreover, the ranges of diverged populations in the marine environment are sometimes difficult to reconcile with modern geography and potential for physical dispersal (Bierne et al. 2003; Cooke et al. 2020; Underwood et al. 2020; Bongaerts et al. 2021), and they may be better understood with reference to historical connectivity, such as during past glacial maxima. A historical perspective may therefore be crucial to understanding gene flow and adaptation in extant populations. However, the value of this approach depends heavily on the temporal resolution of demographic analyses so that their timing can be linked to specific climate events and on the ability to detect and characterize signatures of selection so that these can be used to assess modes of local adaptation.

Emerging techniques based on the sequentially Markovian coalescent (SMC) can be used to reconstruct demographic histories of species in unprecedented detail, potentially revealing links with past climate (Nadachowska-Brzyska et al. 2015; Kozma et al. 2016; Chattopadhyay et al. 2019; Lucena-Perez et al. 2020). However, the most widely used variant of this technique, PSMC (Pairwise Sequentially Markovian Coalescent) (Li and Durbin 2011), has limited power to infer recent events, a problem exacerbated by a large effective population size (Ne) (Schiffels and Durbin 2014). Since corals and many other broadcast-spawning marine taxa have large effective population sizes, most studies so far have focused on changes in the distant past that cover many glacial cycles (Prada et al. 2016; Mao et al. 2018; Fuller et al. 2020; Thomas et al. 2022). Inferences within the timeframe of the most recent glacial cycle require more sophisticated methods such as (Multiple Sequentially Markovian Coalescent) MSMC (Schiffels and Durbin 2014) and SMC++ (Terhorst et al. 2016) that make use of larger datasets (multiple whole genomes) to improve the sampling of haplotypes that share a recent common ancestor.

Even in systems where the effects of past climate change on biodiversity are relatively well understood, the role of natural selection and adaptation in response to climate change remains uncertain (Nogués-Bravo et al. 2018). Addressing this gap for climate-sensitive taxa such as corals is a pressing issue (Torda et al. 2017) directly relevant to their conservation and management in the Anthropocene. Adaptive evolution in corals is complex because it is likely to involve selection on the coral hosts themselves, as well as selection on and/or exchange of their dinoflagellate photosymbionts. Symbiont exchange is of particular interest because it may enable corals to adapt rapidly to anthropogenic climate change (Berkelmans and van Oppen 2006; Torda et al. 2017). Numerous studies have observed variation in host-symbiont associations along environmental gradients (Bongaerts et al. 2013; Camp et al. 2020; Ros et al. 2021), and experiments have demonstrated that a switch in symbiont partnership can be induced by stress (Matsuda et al. 2022). Another potential mode of climate adaptation in corals is selection on the coral host. A range of studies examining population genetic and gene expression differences between heat-adapted and naive corals all suggest that adaptation to heat is likely to involve many loci (Palumbi et al. 2014; Dixon et al. 2015; Fuller et al. 2020; Thomas et al. 2022). Modeling efforts have also attempted to describe the envelope of population genetic parameters and rate of climate change under which corals could adapt based on natural selection (Matz et al. 2018). So far, however, there are few studies (see Smith et al. 2022) that identify signatures of selection in relation to adaptation and survival over a sustained period of warming, such as the transition from the LGM to today.

In this study, we used a population whole-genome sequencing approach to understand the impacts of past climate change on the widespread reef building coral, *Acropora digitifera,* in northwestern Australia. In this region, *A. digitifera* is common on offshore atolls at the shelf-edge and also forms part of a diverse inshore (IN) community (in the Kimberley region) that thrives despite extreme heat, frequent aerial exposure, and highly variable turbidity (Richards et al. 2015; Richards et al. 2019). Modern coral reefs in the Kimberley were extirpated during the LGM, while those offshore may have persisted but would have experienced a period of much reduced shallow-water habitat and been much closer to the coast (Wilson 2013; Solihuddin, O’Leary, et al. 2016; McCaffrey et al. 2020). The contrasting biogeography of these sites provides an ideal case study of the effects of climate change during the last glacial cycle, and our analytical approach is designed to investigate this comprehensively. We do so through demographic modeling based on multiple whole genomes, providing accurate inferences in the window leading up to and following the LGM (1kya—100kya), and through the sensitive detection of signatures of recent selection via extended haplotype homozygosity and population branch statistics. In addition, we use non-host reads to profile the dinoflagellate symbionts inhabiting each coral colony based on standard markers such as the internal transcribed spacer (ITS2) region of ribosomal RNA as well as via mitochondrial sequences and a novel *k*-mer-based distance metric. This combination of approaches allows us to examine the interplay between demographic change, connectivity, selection, and shifts in symbiont community composition during a rapid climate change event for the first time in coral.

## Results

Whole-genome sequencing of 75 *Acropora digitifera* colonies from three reef systems in northwestern Australia yielded a mean per-sample coverage of 19.5X which we used to call approximately 9.6 million high-quality biallelic single nucleotide polymorphisms (SNPs) with GATK (supplementary fig. S1, Supplementary Material online and supplementary table S2, Supplementary Material online). Of the few coral whole-genome studies conducted to date, most (Shinzato et al. 2015; Cooke et al. 2020; Thomas et al. 2022) adopted a shallow sequencing approach (except see Fuller et al. 2020). The relatively high sequencing depth in our study allowed us to reliably call genotypes at more than 95% of sites in 90% of samples (supplementary fig. S1, Supplementary Material online), supporting population-based haplotype phasing with SHAPEIT (Delaneau et al. 2012). As SHAPEIT infers missing genotypes based on phasing information, we tested its accuracy by removing genotypes with high-quality calls and then comparing their original value with that imputed by SHAPEIT. This confirmed that imputation (and by extension, phasing) was generally highly accurate, relatively unaffected by minor allele frequency, but slightly better for sites with fewer missing values and for homozygous genotypes (supplementary fig. S2, Supplementary Material online).

### Population Structure in the Coral Host

**Fig. 1.**
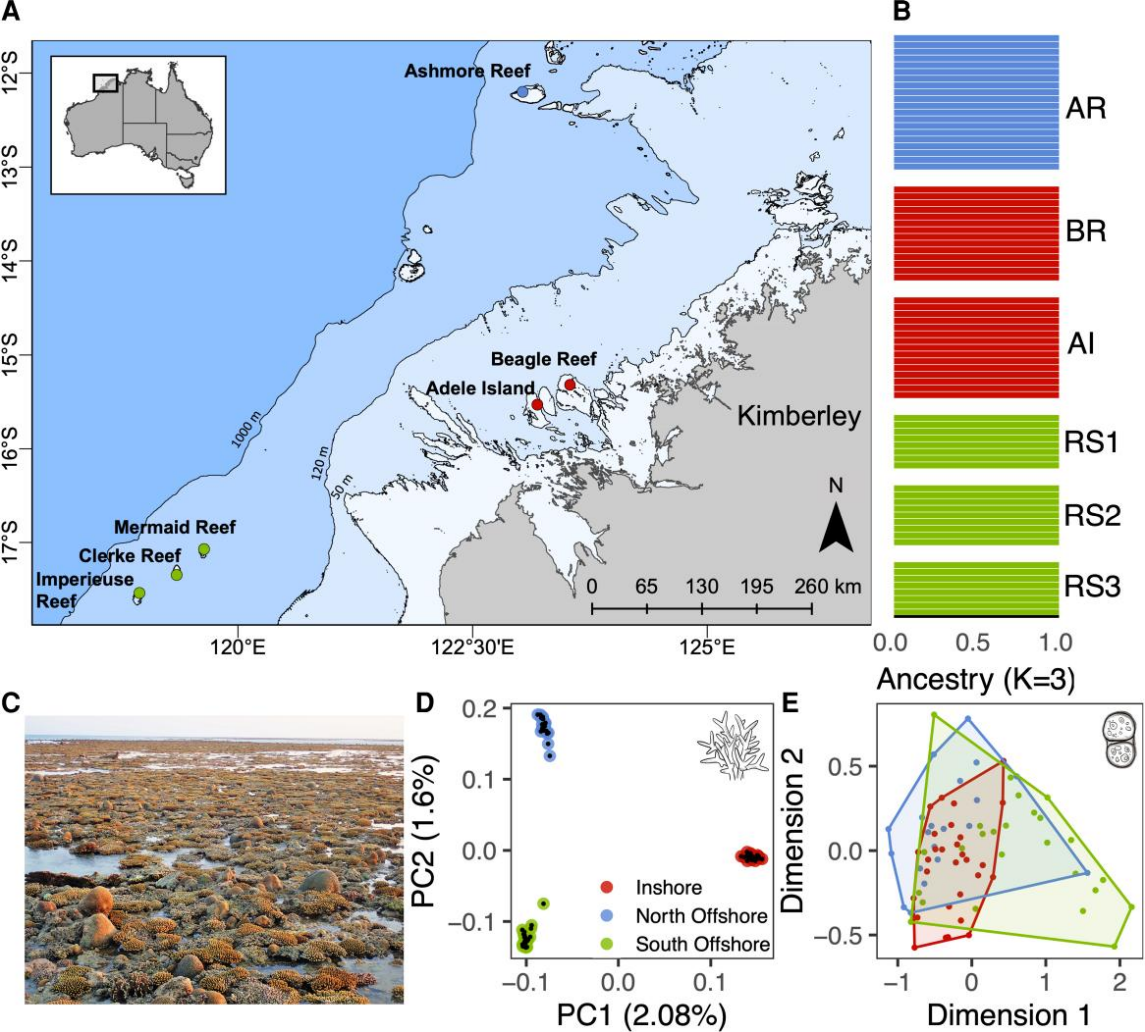
Sampling locations and genetic structure for the coral host and symbionts. All plots use the same color scheme for locations shown visually in panel (B) and described as follows; North offshore, Ashmore Reef (AR) is shown in blue, inshore locations, Adele Island (AI) and Beagle Reef (BR) are shown in red, south offshore locations, Rowley Shoals (RS1: Mermaid Reef, RS2: Clerke Reef, RS3: Imperieuse Reef) are shown in green. (*A*) Sampling locations in the Kimberley region, northwestern Australia. Bathymetric contours are shown at 50, 120, and 1000 m depth with the present day landmass shown in gray. (*B*) Admixture proportions for each colony calculated using ADMIXTURE with K = 3 and colored by the dominant cluster in each location. Each horizontal bar represents a single coral colony. (*C*) Photograph of the reef flat at AI showing corals exposed at low tide. Subaerial exposure for up to three hours during spring low tide is a characteristic feature of the inshore locations, AI and BR in this study. (*D***)** PCA showing the first and second principal components of genetic variation in the coral host. Points represent individual samples and are colored by location. (*E*) Multidimensional scaling plot showing relative pairwise distances between samples based on shared *k*-mers (d2*s* metric) from reads mapping to the dominant symbiont genus, *Cladocopium*. Convex hulls enclose points representing samples from the same location.

Principal Component Analysis (PCA) and fineSTRUCTURE analysis (fig. 1*C*; supplementary fig. S3, Supplementary Material online) showed clear genetic structure that divided corals from the six sampled reefs into three geographically separated groups, hereafter called North Offshore (NO), which includes Ashmore Reef, South Offshore (SO), which includes all reefs from the Rowley Shoals, and Inshore (IN), which includes two locations within macrotidal coral communities in the Kimberley (Adele Island [AI], Beagle Reef [BR]). Using fineSTRUCTURE, we also identified substructure within the inshore population between samples from AI and BR (supplementary fig. S3, Supplementary Material online), however, the very tight clustering of all inshore samples in PCA analyses (PCs 1–3) indicated that this comprised a relatively minor component of genetic variation, and we therefore focused on the three major clusters for our remaining analyses. Pairwise relatedness estimates based on shared genomic regions that were identical by descent (IBD) clearly partitioned samples into the three major clusters but failed to identify a distinction between BR and AI locations (supplementary fig. S4, Supplementary Material online).

The relative distance between PCA clusters, a tree inferred by fineSTRUCTURE (supplementary fig. S3, Supplementary Material online), another tree based on allele counts at established phylogenetic markers (supplementary fig. S5, Supplementary Material online), and relative amounts of IBD segments indicated a closer relationship between the two offshore populations than between offshore and inshore. Consistent with this, genome-wide estimates of F_st_ were markedly lower (F_st_ ∼ 0.007) between offshore populations than between north-offshore and inshore (F_st_ ∼ 0.02) and south-offshore and inshore (F_st_ ∼ 0.02). Despite low overall divergence (as measured with genome-wide F_st_) between these populations, admixture coefficients (calculated using ADMIXTURE; Alexander et al. 2009) showed complete assignment (>99%) of each individual to its parent cluster (fig. 1*B*), suggesting that migration is rare or non-existent between locations. Demographic modeling with fastsimcoal2 (see below) confirmed this as it supported a model with recent gene flow but with very low migration coefficients (probability of migration/individual/generation ∼1e^−4^; supplementary table S8, Supplementary Material online). Analysis of simulated data under this model with ADMIXTURE produced the same complete assignment to locations as observed for the real data (supplementary fig. S16, Supplementary Material online).

To place these western Australian populations in a broader context, we downloaded publicly available whole genome sequencing data from five *A. digitifera* colonies sampled from Okinawa, Japan (NCBI Bioproject PRJDB4188; Shinzato et al. 2015) and for which the sequencing depth was similar to that of our study (16–19x). Using allele counts at established genome-wide markers for phylogenetic inference in *Acropora* (Cowman et al. 2020), we built a phylogenetic tree (using a polymorphism-aware model, HKY + P, in IQ-TREE) that included western Australian and Japanese *A. digitifera* as well as outgroup species *A. millepora* and *A. tenuis* (supplementary fig. S5, Supplementary Material online). This placed all *A. digitifera* populations within the same clade and placed the Japanese samples outside those from Western Australia. The longest branch lengths within the *A. digitifera* clade were around 40-fold shorter than those between *A. digitifera* and *A. millepora*. Consistent with this relatively low divergence between *A. digitifera* populations, we also found that all four shared a single dominant mitochondrial haplotype (supplementary fig. S6, Supplementary Material online), with few samples showing any variation from it. We also found that when a conventional phylogenetic approach (ignoring allele frequency shifts) was used for the same markers, it was unable to resolve differences between western Australian or Japanese populations, or the published *A. digitifera* reference genome (supplementary fig. S7, Supplementary Material online). All four populations are therefore likely to be conspecific and congruent with the published *A. digitifera* genome.

### Symbiont Profiles

Based on the relative proportion of reads classified as Symbiodiniaceae by Kraken (Wood and Salzberg 2014), all samples from all locations were dominated by symbionts from the genus *Cladocopium* (supplementary fig. S8, Supplementary Material online), which is the most common and diverse genus of symbiont in Indo-Pacific corals (LaJeunesse et al. 2018). To investigate the symbiont diversity within *Cladocopium*, we used three complementary approaches, all of which indicated that there was little difference in symbiont composition between locations. First, a haplotype network based on consensus mitochondrial sequences (supplementary fig. S9B, Supplementary Material online) for 41 samples where there was sufficient data (at least 20X mapping depth at mappable sites) revealed that all but one of the 41 samples were dominated by a single haplotype. This represents a much lower level of diversity than was observed in a previous study using the same approach to profile symbionts in *A. tenuis* on the Great Barrier Reef (GBR) (Cooke et al. 2020). Since mitochondrial genomes are rarely used to profile Symbiodiniaceae (Waller and Jackson 2009; Gagat et al. 2017), and cannot easily be linked to known types, we also mapped the putative symbiont reads to the more-commonly used phylogenetic marker of ITS2 sequences, using the SymPortal database (Hume et al. 2019). This revealed a single ITS2 type profile comprising C40c, C72, C40, and C40e, which occurred in most coral samples (supplementary fig. S9A, Supplementary Material online). Finally, in order to minimize inherent biases in ITS2 or mitochondrial markers, we adopted an alignment-free approach based on analysis of shared *k*-mers (i.e., short sub-sequences of defined length *k*) (Reinert et al. 2009; Chan et al. 2014) in the symbiont reads to calculate a distance measure between all possible pairs of samples (see methods). A Multidimensional Scaling (MDS) plot based on this metric (fig. 1*E*) revealed similar levels of within-location to between-location diversity, confirming that there were no consistent differences in symbiont composition between locations.

### Demographic History and Divergence Times

To explore changes in Ne and to estimate divergence times among the coral populations identified above, we performed demographic modeling using two complementary approaches, SMC++ (Terhorst et al. 2016) and fastsimcoal2 (Excofffier et al. 2021). Translating demographic parameters to real timescales for both approaches requires a mutation rate and generation time. Our chosen value of 5 years for generation time is widely used for *Acropora* (Mao et al. 2018; Matz et al. 2018; Cooke et al. 2020) and reflects its fast growth rate combined with the high mechanical vulnerability of older colonies (Madin et al. 2014). For the mutation rate, we calculated an updated value (µ=1.2e^−8^ per base per generation) based on recently published divergence times (Shinzato et al. 2020). To capture uncertainty in both parameters, we ran demographic analyses with SMC++ using alternative published values for the mutation rate (µ=1.86e^−8^, 2.98e^−8^ per base per generation) and alternative plausible values for generation time (3y, 7y). Variation in these parameters did not result in qualitative changes to the shape of Ne curves but generally led to more recent estimates for key events such as bottlenecks and population splits (supplementary fig. S11, Supplementary Material online).

**Fig. 2.**
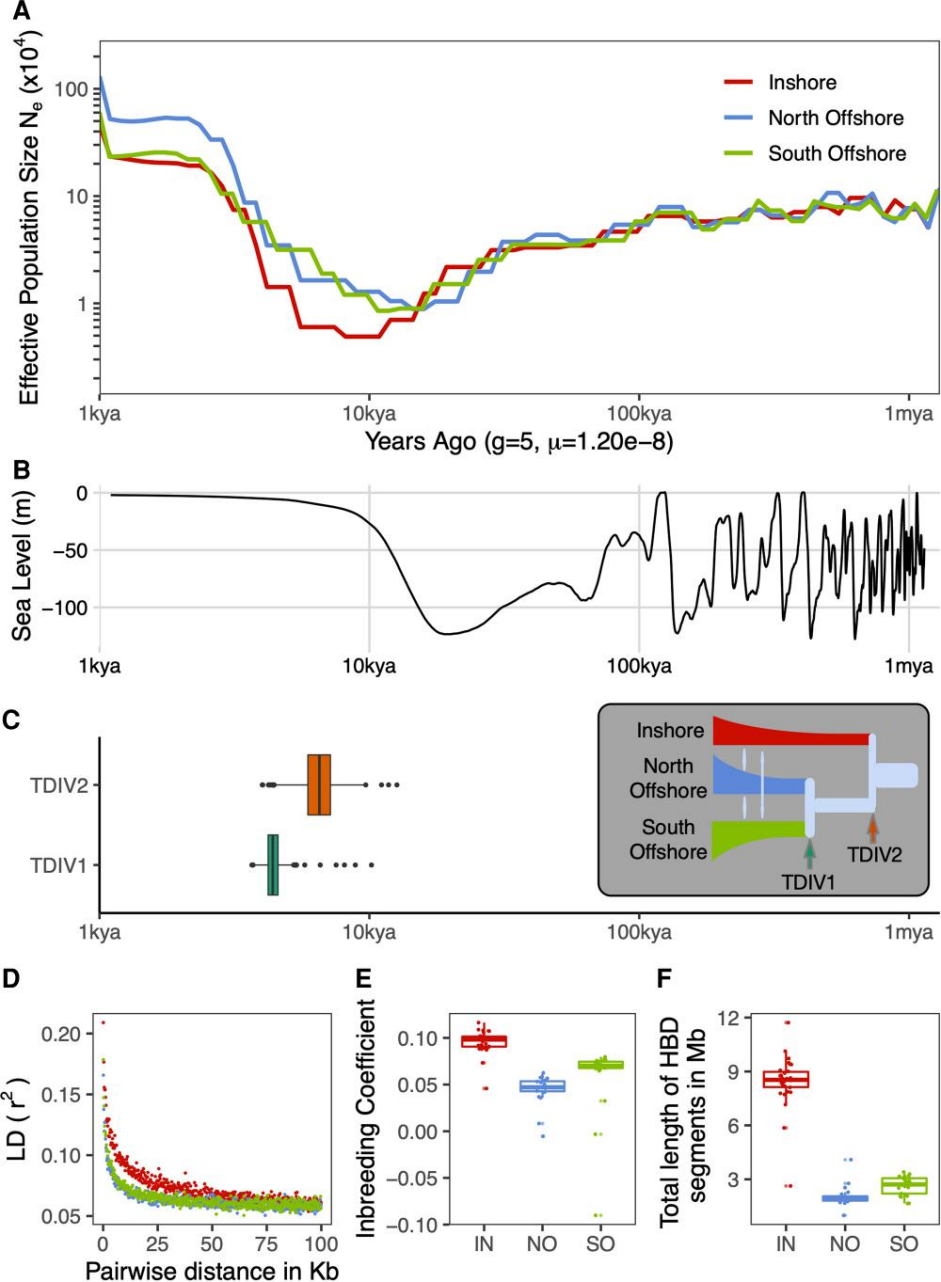
Demographic history of *Acropora digitifera* in Western Australia during the past 1 million years. Locations are denoted by two letter codes, IN, NO, SO, and colored as shown in A. (*A*) Changes in Ne inferred by SMC++. (*B*) Change in global sea level over the same timescale as depicted in A (data from Bintanja and van de Wal 2008). (*C*) Estimated divergence times for the inshore-offshore split (TDIV2) and offshore split (TDIV1) obtained using fastsimcoal2. Inset shows the best model; also used to fit bootstrap parameter estimates. (*D*) LD decay calculated using plink. (*E*) Boxplot of the inbreeding coefficient calculated using plink2 for each sample. (*F*) Total length of genomic regions within each individual that were HBD calculated using ibdseq (Brian L. Browning and Browning 2013). All demographic parameter estimates for both SMC++ and fastsimcoal2 were scaled to real times based on a generation time of five years and an estimated mutation rate of 1.2 × 10^−8^ per base per generation.

Changes in Ne during the past 1 My inferred by SMC++ revealed, qualitatively similar trajectories for the three populations identified in population structure analyses. All experienced a strong bottleneck at some time between 7 and 15 Kya, followed by expansion and stabilization. The timing of these bottlenecks coincides with a period of rapid sea level rise at the end of the LGM (fig. 2*B*). In agreement with the existence of a bottleneck and subsequent population expansion, genome-wide estimates of Tajima’s D for all three populations were negative (supplementary fig. S12, Supplementary Material online).

Populations differed in the timing and severity of the bottleneck, with the strongest and most recent effects seen inshore. This was evident in the SMC++ trajectory as well as the much higher prevalence of homozygous-by-descent (HBD) segments in inshore (fig. 2*F*), along with elevated inbreeding coefficients (fig. 2*E*) and linkage disequilibrium (LD) (fig. 2*D*). Differences between the two offshore populations were less pronounced than between offshore and inshore, however it was clear that the north offshore population retained the highest overall levels of diversity as it had the lowest inbreeding coefficient, smallest proportion of HBD segments and highest SMC++ estimated Ne during the recent stable period (2–5Kya).

Divergence time estimates from both SMC++ and fastsimcoal2 indicate a recent split for all three populations that coincides with the same post-glacial time window as bottlenecks observed in SMC++ analyses. Bootstrap estimates for the inshore-offshore split based on the best-fitting model in fastsimcoal2 (fig. 2*C*; supplementary table S8B, Supplementary Material online) were older (5–8Kya) than those between offshore locations (4–5Kya), matching our expectations based on pairwise F_st_ values and population structure analyses (see above). Estimates from SMC++ were in approximate agreement with this (9Kya) but did not differentiate between inshore-offshore and offshore-offshore splits.

In addition to estimating split times, we used fastsimcoal2 to test a range of competing demographic scenarios (supplementary fig. S14, Supplementary Material online). The results indicate that a model IMc (fig. 2*C* inset) with constant migration between offshore populations and secondary contact between inshore and offshore provides a better fit to the SFS than competing models with strict isolation (SI), ancient migration (AM) or continuous migration (IM) (supplementary fig. S8B, Supplementary Material online). Support for a model (IMc) with contemporary migration was surprising given the lack of evidence for gene flow in admixture analyses but is reconciled by the fact that estimated migration rates from the IMc model were extremely low (∼1e^-4^), (supplementary fig. S8B, Supplementary Material online). To confirm that the IMc model is consistent with this and other key features of our data, we calculated summary statistics and performed admixture analyses for simulated data under this model. These analyses (summarized in supplementary fig. S16, Supplementary Material online) showed similar patterns of HBD, inbreeding coefficient and admixture to our results based on sequencing (fig 1) but produced positive values for Tajima’s D (negative in our real data). This discrepancy in Tajima’s D likely reflects the fact that our simple IMc model was unable to perfectly fit the shape of the 2D SFS at low-medium MAF values (supplementary figs. S18 and S19, Supplementary Material online), a region that has strong effects on Tajima’s D. It also highlights the fact that our demographic models did not capture all factors influencing the SFS, potentially including selection across many linked loci or unmodeled bottleneck effects (Gattepaille et al. 2013).

As our estimates of gene flow assume a constant rate across the genome, we also considered the possibility that gene flow was much higher than estimated and that the observed strong population structure was due to barrier loci that 1) maintained ancient divergence (Tine et al. 2014) or 2) enabled divergence under gene flow via spatially or ecologically variable selection (Malinsky et al. 2015; Rippe et al. 2021). We failed to find evidence for either scenario. The first (barrier loci maintaining ancient divergence) is inconsistent with recent divergence times estimated independently by SMC++ and fastsimcoal2, extremely low admixture coefficients (fig. 2*B*), and the relative rarity of strongly segregating loci in pairwise SFS plots (supplementary fig. S18, Supplementary Material online). Under the second scenario, putative barrier loci should be associated with both high relative divergence (F_st_) and elevated absolute divergence d_xy_ (Cruickshank and Hahn 2014; Malinsky et al. 2015). Although we did find a slight increase in d_xy_ in regions of high F_st_ for inshore versus offshore comparisons, the magnitude of this change was small (supplementary fig. S20, Supplementary Material online), indicating that genomic islands were unlikely to be the primary driver of population structure in *A. digitifera* from Western Australia.

Strong bottlenecks and low migration are both potential contributors to population differentiation. To estimate the relative contribution from these factors, we ran simulations based on the IMc model, but with bottlenecks removed by setting a constant Ne (equal to the ancestral value) and other parameters, including split times and migration rates, set to their best-fitted values. Compared with simulations under the full model, removing the bottleneck dramatically reduced pairwise F_st_; by fivefold for the inshore-offshore split and 2.5-fold for the split between offshore locations (supplementary fig. S17A, Supplementary Material online).

### Genome-wide Scan for Selective Sweeps

**Fig. 3.**
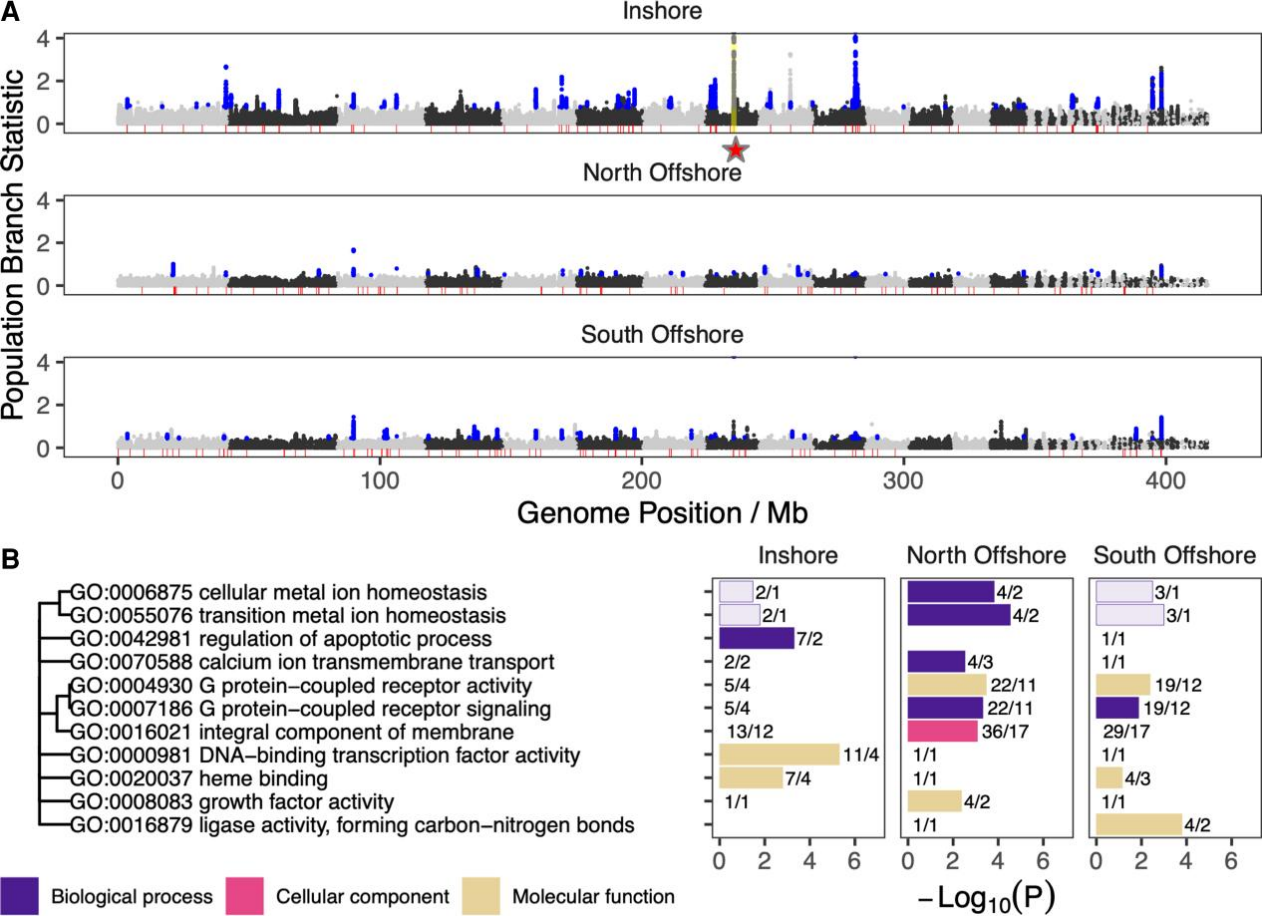
Genome wide distribution of signatures of selection and functional enrichment for overlapping genes. (*A*) Manhattan plots showing values of the PBS and regions under selection identified by EHH based scans. PBS estimates are shown as points for each population and represent allele frequency change since its divergence from the other two. Points are shown in black and gray to indicate transitions between alternating pseudo-chromosomes via mapping to the *A. millepora* assembly from (Fuller et al. 2020). The red shaded baseline shows the location of regions identified as candidates for positive selection using EHH-based scans. Blue points indicate PBS values with probability of false discovery less than 1% under the best fitting demographic model, and which are coincident with EHH scans. Yellow highlighted region (also indicated by a red star) in inshore shows the location of the peroxinectin locus. (*B*) GO term enrichment for regions under selection in inshore and offshore populations. Bar color indicates one of three broad ontologies, BP: biological process, CC: cellular compartment, and MF: molecular function. Relationships between enriched terms based on numbers of shared genes are shown as a dendrogram (left). Length of bar indicates the log odds of enrichment (−Log10(*P*)) based on *P*-values calculated from Fisher’s exact test. Numerical labels indicate the number of genes putatively under selection followed by the number of loci intersected by those genes. Dark shaded bars show significant enrichment based on numbers of genes and numbers of independent sweeps while light shaded bars are significant based on numbers of genes but not sweeps.

To investigate the effects of natural selection on the *A. digitifera* populations identified above, we performed a genome-wide scan for signatures of selective sweeps (regions of low diversity arising due to positive selection and linkage to a beneficial allele). As the primary basis for this scan, we used three statistics (iHS, XP-EHH, XP-nSL) that summarize patterns of extended haplotype homozygosity (EHH) because these have high power to detect selective sweeps within independent populations (iHS) (Voight et al. 2006) or as a contrast between pairs (XP-EHH; XP-nSL) (Sabeti et al. 2007; Szpiech et al. 2021). Following standard binning and normalization practice (see methods; Szpiech and Hernandez 2014), we identified a total of 231 loci (50 kb windows) in which at least one of these three statistics was significant (top 1%) based on the frequency of occurrence of SNPs with extreme values. These putative sweep loci were spread throughout the genome (fig. 3*A*; supplementary table S4, Supplementary Material online) and included 72 specific to inshore, 80 to south offshore, and 79 to north offshore. They were also enriched in SNPs for which the allele-frequency-based indicator of selection, population branch statistic (PBS), had extremely high values (fig. 3*A*).

To control for demographic effects such as bottlenecks, we used simulated data under the best-fitting (IMc) demographic model to calculate threshold values for the PBS that would result in fewer than 1% false positives. As expected, given its more severe bottleneck, this threshold was higher for inshore (IN:0.76) compared with offshore populations (NO:0.48, SO:0.44). Even at this higher threshold, however, the inshore population had more sweep regions identified by EHH statistics that also overlapped SNPs with significant PBS values (33/72, 45%) compared with the north offshore (18/79, 23%) and the south offshore (25/80, 31%).

Of the 1015 genes that overlapped with loci putatively under selection (231 loci identified via EHH-stats; see above), 515 could be assigned a GO term using InterProScan 5 (Jones et al. 2014) based on gene family membership inferred from the presence of conserved domains. Analysis with topGO revealed a total of 11 GO terms across all three ontologies (6 MF; 5 BP; 1 CC) that were enriched (*P* < 0.005; at least two distinct sweep regions) in these genes (supplementary table S5, Supplementary Material online) compared with the background (supplementary table S9, Supplementary Material online) in one or more of the three populations (fig. 3*B*). Since multiple genes often overlapped with each sweep region, we also calculated enrichment statistics based on sweep regions rather than genes as independent units and found that all these terms were also enriched (Fisher’s exact test *P* < 0.005) in at least one population under this criterion (fig. 3*B*).

Three groups of GO terms showed exclusive enrichment in either inshore or offshore locations, potentially reflecting broad patterns of selection related to contrasting environmental conditions. Terms related to membrane G protein-coupled receptors (GO:0004930, GO:0007186, GO:0016021) were strongly enriched in both offshore populations but not in the inshore, with genes underpinning this pattern distributed across 23 independent sweep regions. Exclusive enrichment in inshore was observed for the GO terms, transcription factor activity (GO:0000981) and regulation of apoptotic process (GO: 0042981). Genes supporting enrichment of transcription factor activity in inshore included a diverse range of transcription factors, including those containing homeobox, C2H2 zinc finger, T-box, and forkhead domains, all of which are involved in regulating early development. Enrichment for the GO term, apoptotic process was supported by two independent sweeps, one containing a Bcl-2-like protein (IPR026298) and another that hosted a cluster of 6 genes, each containing a single death effector domain (IPR001875).

### Selective Sweep at the Peroxinectin Locus

**Fig. 4.**
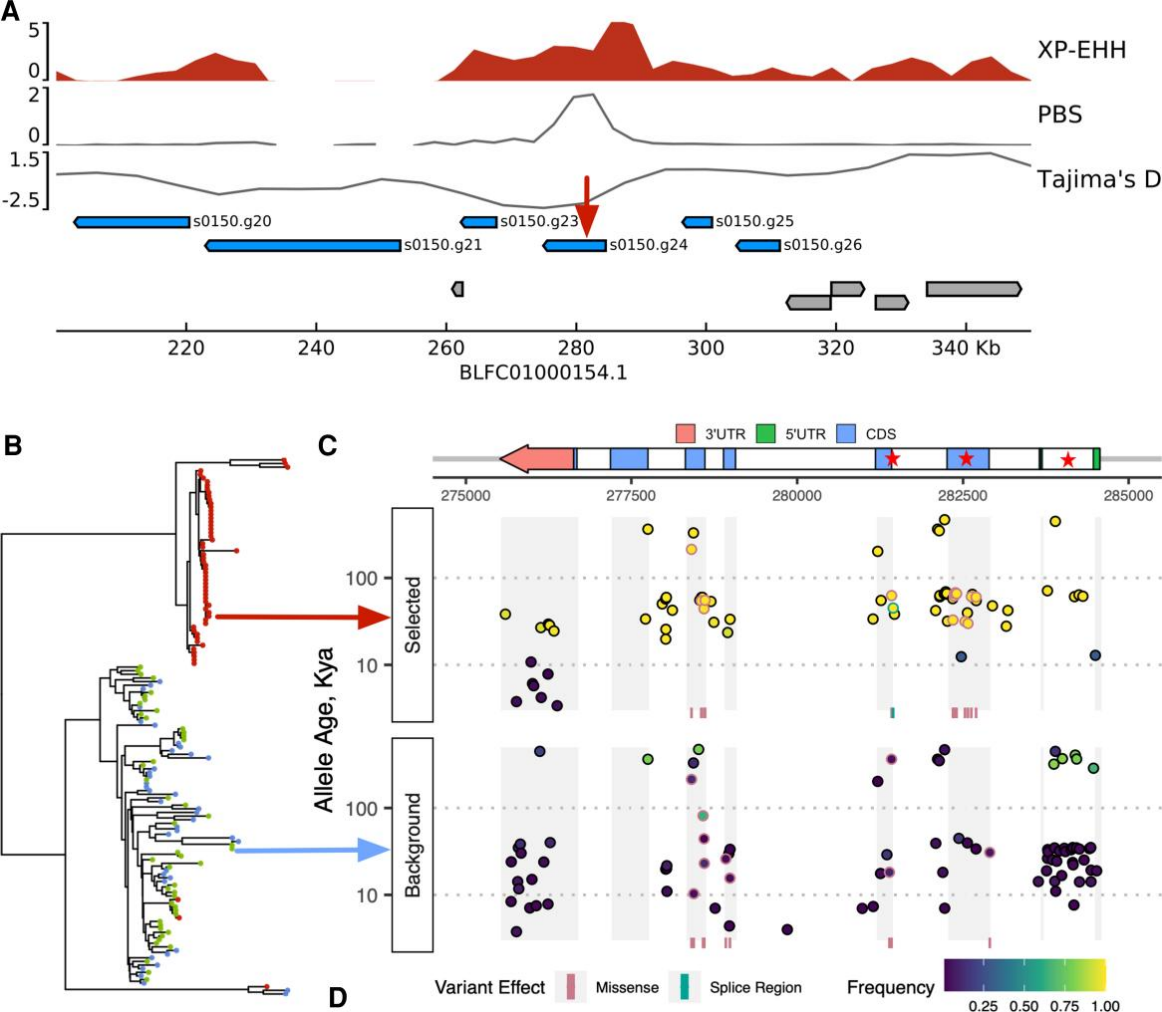
Gene arrangement, haplotype structure and timing of selection for a selective sweep at the peroxinectin locus. (*A*) Zoomed detail at the locus highlighted in yellow in fig. 3*A*. Tracks show values for XP-EHH, PBS and Tajima’s-D for the inshore population. Horizontal bars show the location of genes with peroxinectins in blue and all other genes in gray. (*B*) Neighbor joining tree (left) based on core haplotypes. Core haplotypes include 200 phased variant sites centered on position 281245 on scaffold BLFC01000154.1 (shown with a red arrow in A). Each haplotype is shown as a terminal branch in the tree and colored according to sample location. Haplotypes with the derived allele at the focal SNP all partition into the top clade (selected haplotypes) and those with the ancestral allele into the bottom clade (background). (*C*) Gene structure of s0150.g24 showing exons, cds, and untranslated regions. Stars indicate key regions of the gene mentioned in the text. From right to left they are; first intron, third exon and splice region. (*D*) Age, consequence and frequency of variants overlapping the gene s0150.g24. Scatterplots show variants on selected haplotypes (top) and background haplotypes (bottom). Point positions reflect genomic coordinate (x-axis) and age (y-axis). Point fill color shows allele frequency calculated as the proportion of haplotypes with the derived allele in the given population grouping, i.e selected or background. The position of missense and splice region variants is shown with vertical lines in a strip beneath each scatterplot. Both vertical lines and scatterplot borders for these variants are colored according to variant effect category. Gray vertical bars serve as guides to indicate the position of exons.

To investigate the link between selection, climate change, and gene function in additional detail, we chose to focus on one of the strongest signatures of selection in the inshore population. This locus was associated with the highest PBS values (yellow highlight and red star in fig. 3*A*), low Tajima’s D (fig. 4*A*), and had a clear differentiation between selected and background haplotypes (fig. 4*B*). It also contained by far the largest number (84; next-highest, 7) of near-privately fixed SNPs (>90% allele frequency in inshore, absent in offshore), and of these, over 90% were contained within a single gene, s0150.g24.

Unlike many other sweep loci where the diversity of genes makes it difficult to associate gene function with selection, four of the five genes overlapping this 50 kb sweep region encoded peroxinectin-like proteins (Panther subfamily PTHR11475:SF4; CDD cd09823) and these formed part of a cluster of eight peroxinectin genes found within 200 kb of the sweep. A genome-wide search for haem peroxidases (IPR019791), the parent superfamily that contains peroxinectins, revealed a total of 15 in *A. digitifera*, however only one additional peroxinectin-like gene was found outside the peroxinectin locus. All the remaining haem peroxidases were scattered on different scaffolds throughout the genome indicating, that peroxinectins, but not haem peroxidases in general, are co-located. Orthologous genomic clusters of peroxinectins were also present in other *Acropora* species (*A. millepora*, *A. tenuis*; supplementary fig. S21, Supplementary Material online), indicating that the arrangement is at least as old as the crown age of this genus (∼50Mya; Shinzato et al. 2021).

The strongest statistical indicators of selection at the peroxinectin locus are centered on the gene s0150.g24 (fig. 4*A*). An estimate for the timing of selection on this gene based on the inferred time to the most recent common ancestor for selected haplotypes (8.0–8.3Kya; starTMRCA Smith et al. 2018) approximately match the divergence time for IN corals. Examination of the age of individual alleles at SNPs in this gene inferred by GEVA (Genealogical Estimation of Variant Age) (Albers and McVean 2020) showed a pattern consistent with recent selection on ancestral variation. Young alleles (aged less than 15 Kya) had low frequencies in both selected and background haplotypes, consistent with their emergence after the sweep, whereas alleles older than 15 Kya showed a strong shift toward high frequencies in selected haplotypes compared with background (fig. 4*D*; supplementary fig. S22, Supplementary Material online). GEVA estimates the age of a mutation event giving rise to an allele by comparing TMRCA (Time to Most Recent Common Ancestor) estimates for haplotype pairs where the allele is shared (concordant; younger than the mutation) versus those where it is present in one haplotype and not the other (discordant; older). Although this has been shown to give accurate estimates in humans (Albers and McVean 2020), we expect higher error rates in our study due to a relatively low sample size and uncertainty in input parameters such as the Ne.

Examination of the consequences of variants within the gene s0150.g24 suggests that selected haplotypes may encode a change in exon usage. We identified a total of ten missense variants in the third exon in selected haplotypes compared with just one at low frequency in the background. Such an accumulation of variation in an otherwise conserved region suggests that this exon may no longer be expressed. Although more work is required to confirm this, we note that several variants that might encode the change are present, including a change in the splice region between the third intron and fourth exon as well as five variants in the first intron, a region that often contains gene regulatory elements (Chorev and Carmel 2012).

## Discussion

Our results demonstrate rapid divergence in *A. digitifera* from northwestern Australia, resulting in three genetically distinct populations separated by location. Estimated split times of 5–10Kya and similarly timed bottlenecks in all three populations coincide with geological evidence for the post-glacial reestablishment of reef growth on the tops of atolls (Collins et al. 2011) and inshore reefs (Solihuddin, Bufarale, et al. 2016) in this region. Simulations based on our best-fitting demographic model showed that population size changes were a major contributor to overall levels of population differentiation, most likely through increased genetic drift at small population sizes. Limited dispersal indicates that these bottlenecks are likely to represent founder effects arising from post-glacial colonization, and the two factors (low dispersal and bottlenecks) are the main neutral drivers of divergence.

Since many marine taxa have pelagic larvae and large species ranges, it was initially thought that they should exhibit limited or weak population structure (Palumbi 1992; Cowen and Sponaugle 2009). Recent advances in our understanding of larval dispersal in corals and reef fishes have shown that both can be highly variable (Jones et al. 2009), indicating that in specific settings, strong population structure may be present (Underwood et al. 2020). In agreement with this, population structure has now been observed for a range of coral reef taxa (Warner et al. 2015; Lukoschek et al. 2016; Underwood et al. 2018; Thomas et al. 2020; Adam et al. 2022), but the mechanisms giving rise to this diversity remain poorly understood. Our study demonstrates that population structure can arise rapidly (<10 Kya) when dispersal is low, especially if this is combined with the colonization of new habitats, thereby inducing founder effects which enhance drift. Strong selection (as observed in our study) might also contribute to population structure, however, our neutral simulations show that this is not required to account for rapid divergence.

The limited connectivity inferred between locations in northwestern Australia agrees with a growing consensus based on strong genetic structure (Underwood et al. 2009; Thomas et al. 2020; Adam et al. 2022), local recruitment (Gilmour et al. 2013), and limits to larval movement (Gilmour et al. 2009) that reefs in this region are largely self-seeded. This represents a stark contrast to studies of acroporid species on the GBR (Lukoschek et al. 2016; Cooke et al. 2020; Fuller et al. 2020), and the Ryukyu Archipelago (Shinzato et al. 2015). Both *A. tenuis* and *A. millepora* on the GBR form highly connected populations with weak isolation by distance structure, over hundreds to thousands of kilometers along north-south stretches of the reef (Lukoschek et al. 2016; Matias et al. 2022). Where highly differentiated populations do exist (e.g., *A. tenuis*; Cooke et al. 2020), they show signs of recent admixture and likely reflect ancient splits that are now in secondary contact. This high level of connectivity most likely reflects the fact that reefs in the GBR form a continuous chain with a spacing between 1 and 50 km (Almany et al. 2009), and those in the Ryukyu are connected by the Kuroshio current (Shinzato et al. 2015). In contrast, reefs in western Australia are relatively isolated on offshore atolls or inshore islands separated by distances of 100's of kms (Wilson 2013). The results of this study therefore highlight the potential for physical distances combined with a lack of intermediate habitats to act as a barrier to gene flow, even in a broadcast spawning marine species with a pelagic larval stage. It also underscores the importance of historical context and demographic modeling when interpreting measures of genetic differentiation such as F_st_. In this case, low F_st_ did not mean high connectivity as in Wright’s Island model (Wright 1931), but was revealed to be due to recent divergence via demographic modeling.

Recent work has also shown that the low levels of divergence between northwestern Australian *A. digitifera* populations also extends to southern inshore sites (Ningaloo reef) (Adam et al. 2022), which suggests that *A. digitifera* recolonized Western Australia from a single refuge population after the LGM. Low inbreeding coefficients and higher Ne estimates for the north offshore population are consistent with a refuge at Ashmore reef or recolonization via Ashmore reef from neighboring Indonesia.

Coral spawning in Western Australia takes place primarily in autumn, with a second smaller event in spring. *Acropora digitifera* is among the majority of corals that spawn in autumn (Gilmour et al. 2016), a time when the Leeuwin Current, a poleward-flowing ocean boundary current, is at its strongest, and the potential for current-mediated larval dispersal is at its highest (Feng et al. 2003). This suggests that although the levels of gene flow in our study are low relative to highly connected environments such as the GBR, they may be at the upper end of the spectrum of gene flow for corals in western Australia. A recent study on the spring spawning lineage of *A. tenuis* identified strong population structure (F_st_ > 0.25) separating Rowley Shoals and Scott Reef. Although divergence times have not been estimated for *A. tenuis* populations in WA, previous microsatellite work has shown that the species comprises two deeply diverged spawning lineages (Gilmour et al. 2016; Rosser et al. 2020). Shallower divergences between sites, including between inshore and offshore locations, exist within lineages and have been interpreted as arising due to recolonization after the LGM (Rosser et al. 2020). This suggests that the high F_st_ dividing Rowley Shoals and Scott Reef (Thomas et al. 2022) has arisen rapidly (since the LGM), which points toward even lower levels of gene flow in the spring spawning *A. tenuis* lineage than in autumn spawning *A. digitifera*.

### Contrasting Selection Between Inshore and Offshore Habitats

We identified clear evidence for selection across a wide diversity of loci in all three populations, but with the strongest signals observed in the inshore. The inshore reefs of northwestern Australia are notable for their extreme temperatures (short-term maxima of 37°C), frequent aerial exposure at low tide, and highly variable turbidity (Wilson 2013; Solihuddin et al. 2015). The complex, polygenic nature of these stressors, combined with the fact that signatures of selection often cover many genes (due to linkage) makes it difficult to identify causal alleles or genes (Dixon et al. 2015; Fuller et al. 2020; Smith et al. 2022; Thomas et al. 2022). As more studies document the effects of natural selection on coral populations, it may be possible to identify gene families or pathways that are frequent targets of directional or balancing selection. Our finding that genes involved in regulation of apoptosis were enriched in selective sweeps unique to the inshore population is similar to a pattern observed by Thomas et al. (2022) where genes encoding NACHT and Tumour Necrosis Factor (TNF) receptor domain-containing proteins were identified on two of four linkage groups under balancing selection between reef slope (cooler) and lagoon (warmer) habitats in *Acropora tenuis* populations at the Rowley Shoals. Much remains unknown about the complex apoptotic pathways of corals (Moya et al. 2016), however, there is evidence that they play a role in bleaching (Tchernov et al. 2011) and responding to stress (Cziesielski et al. 2019). However, in the context of inshore corals in the Kimberley, the fact that we also observed enrichment for transcription factors involved in early development suggests that co-enrichment for apoptotic regulators might also be part of a broader suite of selective pressures related to larval development, metamorphosis, and early growth.

In our study, we identified a highly localized signal on a gene (s0150.g24) within a locus dominated by other genes from the same family (peroxinectin-like haem peroxidases). This provides a rare instance in which a gene family targeted by selection is relatively unambiguous. Peroxinectins are best characterized in arthropods where they mediate the immune response via cell adhesion (Johansson et al. 1995) and prostaglandin synthesis (Park et al. 2014). Heat stress experiments in molluscs (Lang et al. 2009), and corals (Voolstra et al. 2009; Shinzato et al. 2021; Traylor-Knowles et al. 2021) consistently identify peroxinectin-like proteins as differentially expressed, and there is evidence that they have undergone recent expansion in some heat-tolerant coral lineages (Shinzato et al. 2021). Unfortunately, the role of peroxinectins in corals has been obscured because many peroxinectin-like proteins are annotated as peroxidasin homologues in the NCBI nr database. For three key publications (Voolstra et al. 2009; Shinzato et al. 2021; Traylor-Knowles et al. 2021), we manually checked sequences annotated as peroxidasin-like and that were differentially expressed in response to heat stress and found that in all cases, the corresponding protein sequences had a similar domain structure to the peroxinectins identified in this paper. All contained one or more characteristic conserved domains of peroxinectins (Panther subfamily PTHR11475:SF4 or CDD cd09823) but lacked the N-terminal leucine rich repeats and immunoglobulin domains found in peroxidasins.

Our results highlight the potential importance of peroxinectins in adaptation to the extreme conditions experienced by inshore corals and invite future work to characterize the evolution and function of co-located peroxinectins in *Acropora* and related taxa. Since the selected haplotypes differ in amino acid sequence from the background, further functional genetic work has a strong chance of identifying the precise nature of the beneficial change, thereby providing a rare opportunity to associate gene function with local adaptive benefit in a wild population.

### Implications for Coral Reefs under Future Climate Change

Our results document the dynamic population responses of *Acropora digitifera* to past climate change. They suggest that this species was likely extirpated throughout much of western Australia during the LGM, but recolonized and underwent rapid population expansion when conditions became favorable. Signatures of selection in all three populations indicate that dispersal and diversification were also accompanied by local adaptation via selective pressure on many loci. Of particular interest in the context of future climate change are the inshore Kimberley populations as these corals are known for their ability to survive extreme heat, turbidity, and exposure (Richards et al. 2015; Richards et al. 2019). The complex selective pressures resulting from future climate change are difficult to predict, however, there is little uncertainty about the fact that corals will need to adapt to higher temperatures. Understanding the genetic basis for this trait is a key prerequisite for assessing the capacity of corals to adapt. Our finding of strong selection on a peroxinectin gene in the inshore adds weight to existing evidence (Voolstra et al. 2009; Shinzato et al. 2021; Traylor-Knowles et al. 2021) that this may be a key gene family in adapting to heat stress. Moreover, we found that peroxinectins are located in a conserved cluster in corals and therefore expect that variation at this locus may be important in determining the capacity of corals to adapt to climate change.

Identifying the origins of population structure is an essential precondition for understanding the relationship between simple measures of divergence such as F_st_ and connectivity. We found that *A. digitifera* populations in northwestern Australia diverged recently, and that gene flow was particularly low between inshore and offshore sites. Connectivity (and gene flow) in coral populations is a key deciding factor in their ability to adapt to climate change (Matz et al. 2018) because it allows natural selection to act on a larger overall gene pool, and because it mitigates against local losses. This combination of risk factors (bottlenecks and low connectivity), seen in our study may also be present in other coral reef systems with similar biogeography such as widely spaced offshore atolls and island chains. Our results therefore suggest that corals from northwestern Australia and other similar systems may be at a higher risk from climate-related losses than in highly connected systems such as the GBR.

## Materials and Methods

### Sample Collection and Sequencing

Small nubbins of *A. digitifera*, approximately 1–6 cm^3^, were collected in November 2017 (Rowley Shoals, Ashmore Reef, Adele Island, and Beagle Reef) and March 2018 (Rowley Shoals) across our three study locations. DNA extractions were performed by Diversity Array Technology Pty Ltd. (DArT P/L) and the extracted DNA was then sent to the QB3 UC Berkeley sequencing center for whole genome sequencing. Initial sequencing was performed on a single NovaSeq S4 flowcell to obtain ∼3 billion 2 × 150 bp paired-end reads across all samples. Additional sequencing was then performed on a second NovaSeq S4 flowcell for 33 samples because they failed to achieve the target depth of 10x in the first batch. Samples included in the second batch of sequencing were spread across all sites in the study (supplementary table S1, Supplementary Material online) and we did not observe any population structure attributable to batch in fineSTRUCTURE analyses (supplementary fig. S3, Supplementary Material online). One sample from inshore (BR_5_121) was likely mislabeled (see supplementary methods), and we excluded it from population structure, demography, and selection analyses.

### Variant Calling, Quality Control and Haplotype Phasing

After verifying that all samples passed read quality checks with FastQC version 0.11.9 and multiQC version 1.6 (Ewels et al. 2016), we then followed the GATK4 (4.1.9) (McKenna et al. 2010) best practice workflow for germline variant calling. Key workflow steps were as follows; raw reads were first aligned to the *Acropora digitifera* reference genome (Shinzato et al. 2011, 2020) using BWA version 0.7.17 (Li and Durbin 2009) with the BWA-MEM algorithm; duplicated reads were removed using the MarkDuplicates function in GATK. Next, HaplotypeCaller was used to call variants in each dataset and generate a file in the GVCF format. The GVCFs from all samples were consolidated into a GenomicsDB data store using GenomicsDBImport and passed to the joint genotyping tools GenotypeGVCFs.

The initial variant call set was filtered with the objective of minimizing bias while maintaining quality biallelic SNPs suitable for population genomic analysis. Filtering steps involved removal of sites that; 1) were within 5 bp of InDels, 2) failed to meet recommended GATK hard filtering quality thresholds, 3) were located within simple repeats, 4) had more than 10% missing genotype calls, and 5) had read coverage outside expected bounds. After filtering, we obtained 9,656,554 high-quality biallelic SNPs from 75 samples. A summary of the number of missing genotypes in all samples after filtering is provided in supplementary fig. S1B, Supplementary Material online. The read-aware phasing mode of SHAPEIT v2 (Delaneau et al. 2012) was used to phase all segregating sites in the filtered VCF file. Additional details are provided in supplementary methods.

### Genome-wide Population Genetic Statistics

Nucleotide diversity(*π*), Tajima’s D, LD, and heterozygosity were calculated genome-wide using the unphased, filtered variant set. The het function in PLINK2 (v2.00a3) (Chang et al. 2015) was used to calculate heterozygosity in each sample. Nucleotide diversity and Tajima’s D were both calculated in 10 kb windows with a 2 kb overlap using VCFtools and VCF-kit (Cook and Andersen 2017), respectively. To avoid bias from gaps and masked regions in these window-based estimates, we used BEDTools v2.29.2 (Quinlan and Hall 2010) to remove windows that have less than 70% of their bases covered, leaving 136,435 windows. Pairwise LD (*r*^2^) was calculated in 1Mb windows using plink v1.9 (Purcell et al. 2007) based on an equal number (20) of samples from each location. Pairwise F_st_ for all SNPs was calculated using the weir-fst-pop function in VCFtools.

### Population Structure

PCA and ADMIXTURE analysis were performed on the unphased, filtered variant set after further filtering to remove sites with a minor allele count of less than or equal to one, or that deviated from Hardy-Weinberg equilibrium (*P*-value < 1e-4). SNPs in high LD were removed using PLINK v1.9 (—indep pairwise 50 10 0.1). PCA analysis was performed using smartpca from EIGENSOFT v6.1.4 (Price et al. 2006) with LD pruned SNPs. Admixture analysis was performed on the same LD pruned data using ADMIXTURE v1.3.0 (Alexander et al. 2009), varying the number of clusters from 1 through to 6. Although the cross-validation error was lowest for K = 1, we chose to use K = 3 because it reflected the number of clusters seen in PCA and because inference of K = 1 is common in situations where overall divergence between clusters is low (Lawson et al. 2012).

We also performed a fineSTRUCTURE (version 4.1.0) analysis (Lawson et al. 2012) on the phased dataset. Inputs were generated by converting SHAPEIT phase files with impute2chromopainter.pl. We assumed a uniform genome-wide recombination rate and allowed the Markov Chain Monte-Carlo (MCMC) to run for 2,000,000 iterations with a burn-in of 1,000,000. Tree inference was performed with 10,000 maximization steps.

Genomic regions inherited by descent (IBD) were identified using the package Refined IBD (Brian L. Browning and Browning 2013). Breaks and short gaps in segments were removed using merge-ibd-segments and pairwise relatedness was calculated based on the total length of shared haplotypes as a proportion of total genome size (Browning and Browning 2011).

### Phylogenetic Inference based on UCE and Exon Probes

To place the *A. digitifera* populations from this study within a broader phylogenetic context, we extracted established phylogenetic markers (ultra-conserved-element and exon sequences from Cowman et al. 2020) from our Western Australian samples, previously published data from Japanese samples (Shinzato et al. 2015) (Bioproject PRJDB4188), and published reference genomes for *Acropora millepora* (Ying et al. 2019) and *Acropora tenuis* (Cooke et al. 2020). First, we mapped the hexa-v2 probeset (Cowman et al. 2020) to the genomes of all three species (*A. digitifera, A. tenuis,* and *A. millepora*) using BWA (v0.7.17). A consensus sequence corresponding to a 1000 bp interval around the central base of each probe was then called using BCFtools (1.11), with ambiguous bases arising from heterozygous sites encoded using their corresponding IUPAC codes. Consensus sequences for Western Australian samples were called based on bam files generated for variant calling. For Japanese samples, raw reads were mapped to the genome using BWA MEM and duplicates marked using GATK, as was done for our own samples. After mapping a total of 16 Japanese samples, we selected five with coverage >15x (DRR099286, DRR099287, DRR099291, DRR099303, and DRR099351). After extracting consensus sequences for all samples, we then used MAFFT (v7.394) (Katoh et al. 2002) to align sequences for each (∼1000 bp) locus separately.

Phylogenetic inference was performed using IQ-TREE (v2.0.3; Nguyen et al. 2015) using 1) a polymorphism (PoMo) aware approach (Schrempf et al. 2016), and 2) a traditional maximum-likelihood approach that ignores allele frequency changes. The allele count file for PoMo was generated using the Fasta2Counts script https://github.com/pomo-dev/cflib based on alignments across all UCE/Exon loci and inference was performed using the HKY + P model with 1000 ultrafast bootstraps. For the traditional phylogenetic approach, we used the same alignments as for PoMo and created a partition file in Nexus format listing them. Using modelfinder (Kalyaanamoorthy et al. 2017), we identified the best model for each partition and used this optimized partition scheme to build a tree with 1000 ultrafast bootstraps (Hoang et al. 2018).

### Demographic History with SMC++

SMC++ analysis was performed based on the unphased vcf call set, including only scaffolds with a length greater than N90 (107,903 bp). The vcf files of each scaffold were converted into SMC++ input format using the vcf2smc script while masking large uncalled regions. Multiple SMC files were generated for each scaffold by varying the choice of ‘distinguished individual’ over all samples. To estimate population size histories, all SMC++ input files were used together in a single run with the options, thinning 3000, 50 EM iterations, 40 knots, mutation rate 1.20e^−8^ per base per generation, and starting and ending time points set to 20-200 000 generations. Divergence times for each population pair were inferred using the SMC++ split command with marginal estimates produced by using the estimate option. To address the uncertainty in SMC++ analysis from mutation rate and generation time parameters, we tested two additional mutation rates: 1.86e^−8^ (Cooke et al. 2020); 2.98e^−8^ (Mao et al. 2018); and three generation times, 3, 5, and 7 years (Oppen et al. 2000; Baria et al. 2012; Matz et al. 2018).

### Demographic History with fastsimcoal2

To prepare data for fastsimcoal2 (Excofffier et al. 2021), we used BCFtools to remove sites located in genic regions and performed LD pruning in 1000 bp windows with a cutoff of r^2^>0.3. After removing sites with missing genotypes, we used easySFS (https://github.com/isaacovercast/easySFS) to generate a joint three-dimensional folded SFS with 257,314 SNPs. To utilize the mutation rate in branch length calculations, we estimated the number of monomorphic sites based on the proportion of mappable sites defined by the SNPable pipeline.

First, we tested four alternative topologies, indicating alternative splitting modes among three populations (Supplementary table S8A, Supplementary Material online). For each model, fastsimcoal2 (version 2705) was used to fit parameters to the joint SFS with 50 ECM optimization cycles and 200,000 coalescent simulations. Model fitting was repeated 100 times based on different randomly sampled starting parameter values. We report the best AIC and likelihood values for all four models (across the 100 runs) in supplementary table S8A. Based on the best fitting tree topology ((NO, SO), IN), we then tested six competing models, all with exponential population size change (Supplementary fig. S13, Supplementary Material online). Model normalized relative likelihoods (Excoffier et al. 2013) (Supplementary fig. S15, Supplementary Material online) supported one of these models (IMc; secondary contact for offshore-inshore but isolation with migration for offshore-offshore). Extended details of the model selection process are provided in supplementary methods. Confidence intervals for the parameters of the best model were estimated using 100 non-parametric bootstraps, each of which was generated by sampling 257,314 SNPs with replacement from the original set of SNPs. For each bootstrapping data set, we performed 20 independent runs. Final results are shown in Supplementary table S8B, Supplementary Material online.

### Analysis of Simulated Data under the Best Fitting Model

We generated simulated data under the best fitting parameter set for the IMc model using fastsimcoal2 with an identical model specification file to that used for SFS fitting. We performed 50 independent simulations, each of which used parameters drawn randomly from a uniform distribution across a 90% confidence interval based on our bootstrap estimates (see above). Each simulation generated 20 scaffolds of length two mb. Based on this data, we then calculated: 1) the length of HBD segments using ibdseq, 2) inbreeding coefficient using plink2, 3) Tajima’s D using vk tajima, 4) admixture coefficients using ADMIXTURE, and 5) population branch statistics using plink. All calculations were performed using identical settings to those used for real data. The results are shown in Supplementary fig. S16, Supplementary Material online.

Simulations based on a modified version of the IMc model were used to assess the contribution of population size changes (i.e., the bottleneck) to population differentiation. The IMc model was modified so that the total population was conserved at its ancestral size, dividing this at population splits to achieve equal populations in the most recent time period. All other parameters were left unmodified. We ran 10 independent simulations using the same process described above with parameter draws allowing variation in divergence times and migration rates but not population sizes. Based on this data, we calculated pairwise F_st_ and performed PCA using plink2. Results are shown in Supplementary fig. S17, Supplementary Material online.

### Signatures of Selection

We used selscan v1.3.0 (Szpiech and Hernandez 2014) with default parameters to calculate test statistics (iHS, XP-EHH, and XP-nSL) based on extended haplotype homozygosity (EHH). Normalization was performed in 50 separate allele frequency bins using the companion program norm. After normalization, SNPs with extreme values were identified genome-wide based on the following criteria (|iHS|>2, XP-EHH/XP-nSL > upper first percentile). We then calculated the proportion of SNPs with extreme values within 50 kb windows and identified windows as candidates for selective sweeps as those in the top 1% based on this proportion. This process was performed separately for each of the three test statistics (iHS, XP-EHH, XP-nSL) and multiIntersectBed (Quinlan and Hall 2010) was used to report the overlapping candidate regions of all tests. Since our goal was to identify sweeps unique to each population, we removed those that were significant based on iHS in more than one population. This was not required for the cross-population tests since those already target regions that differ between populations.

We also calculated population branch statistics (PBS), which measure the change in allele frequency in a focal population since its divergence from two other populations. First, we used the –fst function in PLINK to calculate F_st_ statistics genome-wide for all pairs of populations, using the default F_st_ calculation (Hudson). These F_st_ values were then used to calculate the population branch statistic as described in its original paper (Yi et al. 2010). We then used coalescent simulations based on the best-fitting demographic model to determine separate threshold significance values for PBS in each population (see supplementary methods). Our approach differs slightly from the original usage of PBS since we follow Wang et al. 2018 by allowing the outgroup (inshore in this case) to be the focal population and use simulations to control for false positives.

### GO Enrichment Analysis

To support GO enrichment analysis, we performed functional annotation of *A. digitifera* genes, assigning GO terms via blast and Interproscan searches (see supplementary methods). The R package topGO v2.42 (Alexa et al. 2006) with the default ‘weight01’ algorithm was used to test for enrichment of GO terms assigned to genes within sweep regions. In this analysis, all genes overlapping with putative selective sweeps were assigned to the target set, and the complete set of all annotated genes was assigned as the background set. Since genes are not randomly distributed across the genome, we also performed a second test where GO terms were assigned to sweep-regions and not to individual genes. As this test was used as a complement to the first, we performed it only for GO terms that were significant at the gene level. For the second test, we first assigned GO terms to all 50 kb regions in the genome based on the GO terms assigned to overlapping genes. We then calculated a *P*-value based on Fisher's exact test by counting the number of sweep regions (a subset of all 50 kb regions) with a given term and comparing this to the background count across all regions.

### Symbiont Analysis

Using a custom database composed of the genomes of five common coral associating Symbiodiniaceae genera and the *Acropora digitifera* genome assembly, we classified raw reads from all samples using kraken v1.0 (Wood and Salzberg 2014). This confirmed the dominance of *Cladocopium* in all samples and identified between 4k and 1.7 M (median 260k) reads originating from Symbiodiniaceae. Next, we mapped the reads to the mitochondrial genome of *Cladocopium* C1 and built a haplotype network using PopART (Leigh and Bryant 2015) with the consensus sequences of 41 samples after removing samples with less than 20X average mapping depth (excluding regions with no reads mapped). We also mapped non-host reads to ITS2 sequences from the symportal (Hume et al. 2019) database and quantified their abundance by counting the number of uniquely mapped reads to each ITS2 reference sequence. Finally, we used an alignment-free method (https://github.com/chanlab-genomics/alignment-free-tools) to calculate the d2s metric based on shared k-mers in sequencing reads from each pair of samples. This produced a set of pairwise distances which we visualized using an MDS plot (fig. 1*E*).

Although the d2s metric has previously been shown to discriminate between whole genome sequences of different Symbiodiniaceae species (Dougan et al. 2022), its power to distinguish differences based on low coverage whole genome sequencing has not previously been established. To establish such a benchmark, we used d2s statistics to analyse data from a study of Acropora tenuis samples in the GBR. Although the overall sequencing depth in that study was much lower than ours (approx 2–3x per sample), we found that d2s statistics successfully recapitulated observed differences identified through a mitochondrial haplotype network (figure 2 in Cooke et al. 2020 vs. supplementary fig. S10, Supplementary Material online). This power to detect differences despite low overall coverage is surprising if one considers genome coverage to be uniform. We found, however, that despite having a coverage of less than 0.4x, there were over 3.5 million sites covered by at least one read in at least 40 samples. These regions (likely repeats) provide for shared kmers between samples and thereby provide power even at low overall coverage.

### Estimating the Timing of Selection at the Peroxinectin Locus

We used the R package starTMRCA (commit cf9f021 from github) (Smith et al. 2018) to estimate the timing of selection at the peroxinectin locus. Since we did not know the beneficial allele (required by starTMRCA), we instead identified alleles likely to be in complete linkage with it to serve as its proxy. We did this by choosing sites for which the derived allele was nearly fixed (on all but three haplotypes) in the inshore population and completely absent offshore. There were 84 such SNPs within the sweep locus, of which 75 were found within the gene s0150.g24 that overlapped with the strongest statistical indicators of selection (fig. 4*A*). Of these 75 sites, we chose three spanning the length of the gene (at positions 278594, 281245, and 282923). After performing visual checks of haplotype structure (see supplementary methods) we then ran starTMRCA separately for each of the three chosen SNPs using a 1Mb phased region around the center of s0150.g24. Other parameters included a mutation rate of 1.2e^−8^ per base per generation, a recombination rate of 3.2e^−8^ per base per generation (see supplementary methods), a chain length of 10000, a proposal standard deviation of 20, and an initial value of TMRCA drawn from a uniform distribution from 0–10000 generations. Convergence was checked by running ten independent chains and calculating the Gelman diagnostic using the coda package in R. For each SNP, we recorded the median value of the posterior estimates of the TMRCA after discarding the first half as burn-in. Our final estimate for the time of selection on the locus is reported as the range of estimated values across these three SNPs.

### Estimating Allele age With GEVA

To estimate the time of origin for derived alleles in the peroxinectin locus, we used Genealogical Estimation of Variant Age (GEVA) (Albers and McVean 2020). First ancestral and derived alleles were polarized using est-sfs (Keightley and Jackson 2018) (see supplementary methods). GEVA was run assuming an Ne of 30000, a mutation rate of 1.2e^−8^ per base per generation, and a recombination rate (3.2e^−8^ per base per generation) as used for starTMRCA.

### Phylogenetic Analyses of Haem Peroxidases

To investigate the evolutionary origins of the peroxinectin locus, we used blastp to search for homologous genes in four other coral species, *Acropora millepora*, *Acropora tenuis*, *Porites lutea*, and *Pachyseris speciosa*. Protein sequences for all genes identified as belonging to the haem peroxidase family (IPR019791) by Interproscan were extracted from *Acropora digitifera*. Using these as query sequences, we identified all close homologs (e-value < 1e^-10^) from the protein sets of all other species using blastp. These were then aligned using the MAFFT (v7.394) (Katoh et al. 2002) with the algorithm set to auto. After masking positions with more than 50 missingness, IQ-TREE (v2.0.3; Nguyen et al. 2015) was used to perform tree inference based on this alignment with 1000 ultrafast bootstraps and automatic model selection using modelfinder.

## Supplementary Material

msac201_Supplementary_Data

## Data Availability

**Raw data:** NCBI Bioproject PRJNA805369. **Code and accessory data:** DOI: 10.5281/zenodo.6893999.
