# Supplementary material for: Evolutionary Responses of a Reef-building Coral to Climate Change at the End of the Last Glacial Maximum
Source: Mol Biol Evol. 2022 Oct 11;39(10):msac201. doi: 10.1093/molbev/msac201 (PMC9578555; doi:10.1093/molbev/msac201)
Supplement: msac201_Supplementary_Data [file msac201_supplementary_data.zip › Supplementary materials.pdf]

Sample collection and sequencing

75 samples from adult corals across our three study locations were selected from a larger pool of 564 samples collected as part of a separate study across a wider geographic area that was primarily based on DArT sequencing (Adam et al. 2022). At these three study locations, small nubbins of *A. digitifera*, approximately 1-6 cm3 were collected in November 2017 (Rowley Shoals, Ashmore Reef, Adele Island and Beagle Reef) and March 2018 (Rowley Shoals) across 21 sites and stored in 100% ethanol. These samples were later subsampled to be sent to Diversity Array Technology Pty Ltd. (DArT P/L) for further processing. DNA extractions were performed for all samples by DArT and the remaining DNA (not used for DArT) was sent to the QB3 UC Berkeley sequencing centre for whole genome sequencing. Samples for whole genome sequencing were selected randomly from samples previously sequenced by DArT and after excluding 7 that failed initial quality checks an additional 7 replacement samples were also randomly selected. Initial sequencing was performed on a single NovaSeq S4 flowcell to obtain ~3 billion 2x150bp paired-end reads across all samples. Additional sequencing was then performed on a second NovaSeq S4 flowcell for 33 samples because they failed to achieve the target depth of 10x in the first batch. Samples included in the second batch of sequencing were spread across all sites in the study (supplementary table S1) and we did not observe any population structure attributable to batch in fineSTRUCTURE analyses (supplementary fig. S3).

Identification of mislabelled sample

Initial population structure analyses revealed a single sample (BR_5_121_S125) coded as inshore that clustered with south offshore samples. To check whether this was a genuine example of migration or a mislabelled sample we combined our whole genome sequencing data with all raw reads from the DArT dataset. Raw DArT reads were first mapped to the genome using bwa mem (v0.7.17) and variants called with freebayes (v1.3.2-dirty; (Garrison and Marth 2012)) with min-mapping-quality set to 30 and min-base-quality set to 20. The resulting vcf file was then filtered to retain only variants with maf>0.1, min depth of 8x and min mean depth of 15. This vcf was then merged with the filtered vcf file from whole genome analyses retaining only variant sites common to both approaches. Using this combined vcf we then calculated the relatedness using the relatedness2 statistic implemented in VCFtools (v0.1.16) between all pairs of samples and found that all but two pairs had relatedness values < 0.1. The remaining two pairs had relatedness values (>0.48) indicative of clones or identical samples. One of these pairs was irrelevant to the current analyses as it

concerned two DArT samples only. The remaining pair indicated a match between sample RS3_S_252 from the DArT dataset and BR_5_121_S125 from the WGS dataset indicating that this sample was mislabelled at some point after DArT sequencing, and its true origin was the Rowley Shoals.

Variant calling and filtering

Our variant calling pipeline was implemented using snakemake version 5.5.4 (Köster and Rahmann 2012) and is available online at (https://github.com/bakeronit/snakemake-gatk4-non-model).

The initial variant call set was filtered with the objective of minimising bias while maintaining quality biallelic SNPs suitable for the population genomic analysis. Filtering steps were performed sequentially as follows;

1. Sites within 5bp of InDels were removed using BCFtools version (1.10.2) (Danecek et al. 2021)
2. Hard-filtering thresholds were applied using the GATK VariantFiltration tool based on recommended parameters as follows (QD < 10, QUAL < 30, SOR > 3, FS > 60, MQ < 40, MQRankSum < -12.5, ReadPosRankSum < -8). Abbreviated parameters are QD=QualByDepth, QUAL=Quality, SOR=StrandOddsRatio, FS=FisherStrand, MQ=RMSMappingQuality.
3. Sites located in simple repeat regions identified by mdust version 2006.10.17 were removed (Li 2014).
4. Sites were removed if they had more than 10% missing or low quality genotype calls under the thresholds GQ > 20 and DP >3. (GQ=Genotype Quality; DP = sample read depth). This was performed using VCFtools v0.1.16 (Danecek et al. 2011)
5. Sites were removed if their read coverage fell outside expected bounds (mean per-sample depth less than 8 or greater than 31) because this could indicate collapsed repeats or regions with low mappability.

After all filtering steps, we obtained 9,656,554 high-quality biallelic SNPs from 75 samples. A summary of the number of missing genotypes in all samples after filtering is provided in supplementary fig. S1B.

Haplotype phasing

To resolve haplotype information, we used the software SHAPEIT v2 (Delaneau et al. 2011) which can phase segregating sites in a sample of unrelated individuals. To improve phasing accuracy we also incorporated information from phase informative reads in mapping files (bam format). Phase informative read information was first extracted from bam files using the tool, extractPIRs. Next we used the SHAPEIT assemble command to run the standard population-based phasing together with the read aware phasing module. This was performed separately for each scaffold and the results were combined into a single file in VCF format.

Missing genotypes were imputed by SHAPEIT2 during the assembly run. To evaluate the accuracy of imputation, we performed a "masked analysis" (Verma et al. 2014), in which a subset of genotyped SNPs in the samples was randomly pruned and then imputed as missing data. We compared the imputed genotypes to their original genotypes to estimate the concordance which indicates the performance of imputation with respect to that set of SNPs. A summary of this imputation accuracy check is provided in supplementary fig. S2.

Demographic history with fastsimcoal2

To model demographic history while accounting for population structure, we carried out SFS based demographic modelling using fastsimcoal2 (Excoffier et al. 2021). We used all samples except BR_5_121_S125 as per our SMC++ analysis. To minimise the bias from linkage disequilibrium and selection, we used BCFtools to remove sites located in genic regions and performed LD pruning in 1000bp windows with a cut-off of $r2>0.3$. To utilise the mutation rate in branch length computation, we estimated the monomorphic sites based on the proportional number of mappability sites defined by the SNPable pipeline we used in MSMC analysis. We also filtered out sites with missing genotypes and then used easySFS (https://github.com/isaacovercast/easySFS) to generate a joint three-dimensional folded SFS with 257,314 SNPs.

All the demographic models tested with fastsimcoal2 assume that gene flow (if modelled) is constant across the genome. To check that this assumption was appropriate we plotted the pairwise SFS for each population pair (supplementary figure S18) and checked that there was not a large excess of strongly segregating alleles (high frequency in one population, low in another). This type of SFS pattern (see for example (Tine et al. 2014)) suggests ancient divergence followed by secondary contact with genomic islands resulting from barriers to gene flow. None of the pairwise SFS's for our data exhibited this pattern. We also used the

PopGenome (Pfeifer et al. 2014) package in R to calculate relative pairwise divergence (Fst) and absolute divergence dxy in 50kb windows across the longest 20 genomic scaffolds. If divergence is occurring under high gene flow via barrier loci (eg resulting from local selection) then regions with very high Fst should also be associated with high dxy. The absence of a strong association (supplementary figure S20) is consistent with our broad finding that divergence occurred under low gene flow.

We firstly tried to test which population tree topology the SFS data support without considering the population size changes and migrations. In this step we tested four alternative topologies indicating alternative splitting modes among three populations including inshore split first, south offshore split first, north offshore split first, or a polytomy tree of three populations (supplementary table S8A). For each model, fastsimcoal2 (version 2705) was used to fit parameters to the joint SFS with 50 ECM optimization cycles and 200,000 coalescent simulations used to compute the likelihood. This model fitting process was repeated 100 times based on different randomly sampled starting parameter values. This gave clear support for the inshore split first model as it always had the lowest Akaike information criterion (AIC) value across all 100 runs. We report the best AIC and likelihood values for all four models (across the 100 runs) in supplementary table S8A.

Based on the population tree ((NO, SO), IN), we then tested six competing models all with exponential population size change (supplementary fig. S13). These models were primarily designed to test different migration scenarios and comprised; 1) strict isolation (SI), 2) continuous migration between all demes at all times (IM), 3) continuous migration among three populations only after offshore divergence, ie secondary contact for offshore-inshore but isolation with migration for offshore-offshore (IMc), 4) isolation with recent secondary contact (SC), 5) early migration after offshore divergence (EM), 6) ancient migration between inshore and offshore ancestor followed by strict isolation (AM). We specified the search ranges for the current and ancestral effective population sizes between 1,000 and 1,000,000, and the effective population size for the offshore ancestor to between 100 and 10,000, but with an open upper bound that is extended if parameters get close to the boundary during the ECM optimisation. Divergence times were allowed to vary between 100 and 10,000 generations. The range of migration rates was assumed to be between 10-7 to 10-3 with open upper bounds. For the SC and EM models, we allowed the time of changed

migration (TMIG) to be between 100 generations and the offshore divergence time (using paramInRange).

Parameters for all six models were initially estimated using the same process as outlined above. After parameter estimation, we observed that the SC and EM models were converging towards the IMc model as TMIG kept being pushed to the lower bound (100 generations) in the EM model while being optimised to be close to offshore divergence time in the SC model (supplementary fig. S14). We thus deprecated these two models in the following likelihood estimates and model comparison. Next, we compared different models using the model normalised relative likelihood (Excoffier et al. 2013) (supplementary fig. S15, supplementary table S8B), and estimated the parameter ranges (supplementary table S8B). As a result of this process we chose the IMc model as it had a model normalised relative likelihood of close to 1 whereas this was 0 for all other models. We then estimated confidence intervals for the parameters of the best model using 100 non-parametric bootstrapping datasets, each of which was generated by sampling 257,314 SNPs with replacement from the original set of SNPs. This sampling was performed using the sample tool (alexpreynolds.github.io/sample). For each bootstrapping data set, we performed 20 independent runs. Final results shown in supplementary table S8B show 95% confidence intervals based on the distribution of fitted parameters from these independent runs.

To check the fit of the IMc model to the 2D SFS we plotted the residuals (observed SFS - modelled SFS; supplementary figure S19). Although this showed a good fit across the majority of the 2D SFS we found that the model alternately under and over estimated allele frequencies in alternating bands along the lower left corner. Since the largest of these bands is also visible in the observed SFS (supplementary figure S18A) we used two alternative methods of conversion from vcf to SFS to ensure that it was not due to an artifact of our SFS generating pipeline. Instead of using easySFS to generate the SFS we used the scripts, vcf2sfs, foldSFS and SFSTools.R available on the fastsimcoal website. As input to vcf2sfs we used phased genotypes pruned for LD (same settings as for our easySFS pipeline) with the ancestral allele encoded as the reference (see section estimating allele age with GEVA). Without any additional filtering this resulted in the SFS labelled (vcf2sfs - A) in supplementary figure S18. An alternative SFS (labelled, vcf2sfs - B) was also generated by first removing all sites within 500kb of the top 1% of windows identified as selective sweeps

by either iHS, XP-EHH and XP-nSL. Since the same band is visible in all versions of the SFS we concluded that it is not an artefact of our SFS generating pipeline.

Empirical false discovery rate for signatures of selection based on population branch statistics

We used simulated data under our best-fitting demographic model with fastsimcoal2 to calculate the distribution of population branch statistics (PBS) for each population arising under neutrality. Simulations were performed 50 times using randomly selected values across the bootstrap-estimated 90% confidence intervals for model parameters. Since this generated a much larger number of PBS values to our real dataset, and includes many sites in LD, we randomly selected 100k values from this simulated data and from our real data. The resulting 200k were then ranked by PBS value (0 the highest) and the false positive rate for the ith ranked value was calculated by counting the number of false (ie simulated) values from ranks 0 through to i and dividing this value by 0.5i. We then calculated the threshold value, above which this empirically calculated error dropped below 0.01 (1%) and used this as our criteria for significance. This was done separately for each population.

Mapping to pseudo-chromosomes

We used ragtag v.1.1.1 (Alonge et al. 2019) to align the *Acropora digitifera* genome to the *Acropora millepora* chromosome-level genome assembly (Fuller et al. 2020) with default settings. This placed 735 of the 955 *A. digitifera* scaffolds in pseudo-chromosomes, comprising 97% of assembled bases. We used this mapping to translate between scaffold level and pseudo-chromosome coordinates for the purpose of visualization only. Specifically, it was used to create the Manhattan plot (fig 3A).

Gene annotations

Gene models for the *Acropora digitifera* version 2 assembly were obtained from the authors of its original publication(Shinzato et al. 2020) in gene feature annotation (GFF3) format. As these gene models are based on scaffolds from the original assembly (available at https://marinegenomics.oist.jp/adig/viewer/info?project_id=87) that have not undergone the RefSeq curation process their coordinates needed to be updated to match the ncbi assembly (GCA_014634065.1) that we used for our analyses. To do this we first aligned the two genomes with Cactus (Armstrong et al. 2020) and then used the ucsc chain and liftOver utilities(Kuhn et al. 2013) to generate updated gene model coordinates. The resulting updated

gene models and full details of the lift-over process are available via the online code repository https://github.com/bakeronit/acropora_digitifera_wgs for this paper.

Starting from these updated gene models we first selected the longest transcript per gene using cgat toolkit(Sims et al. 2014) and then extracted nucleotide and protein sequences for each coding sequence using gffread(Pertea and Pertea 2020). Functional annotations for these genes were then obtained by performing blastp and blastx searches on protein and nucleotide sequences respectively against the Swissprot database (downloaded 2021 May 9)(Bairoch and Apweiler 2000), filtering to include hits at e-value < $1e^{-5}$ only. We then selected the best available blast[xp] hit for each gene and assigned this as its closest putative homolog. In addition, we used the Uniprot ID mapping service to look up detailed functional information (including GO terms) for these homologs.

Our initial gene ontology enrichment analysis was performed based on these GO terms assigned based on blast hits to Swissprot, however, we found that this often resulted in enrichment of highly specific gene ontology terms that were clearly spurious as they involved functions that are not present in Cnidarians. To resolve this issue we decided to use GO terms assigned using Interproscan version 5.53-87(Jones et al. 2014), which uses functional information assigned to conserved domains rather than to specific genes. A complete table of annotated genes resulting from both BLAST and Interproscan annotations is provided as supplementary table S9.

GO enrichment analysis

Formal statistical analysis for enrichment of GO terms is challenging because the terms themselves are not independent, and because genes are not randomly distributed across the genome. The R package topGO v2.42 (Alexa et al. 2006) attempts to deal with the first of these issues (non-independence of GO terms) by weighting the assignment of genes to terms in a way that increases the significance of more specific terms at the expense of more biologically general parent terms. We, therefore, used topGO with the default “weight01” algorithm for all enrichment tests. To deal with the second issue (non random distribution of genes across the genome) we calculated enrichment statistics at two levels. First we evaluated enrichment at the gene level. In this analysis all genes overlapping with putative selective sweeps were assigned to the target set and the complete set of all annotated genes was assigned as the background set. Since this analysis ignores the fact that multiple genes from

the same GO term might be present in the same sweep region we also performed an enrichment test based on sweeps rather than genes. As this test was used as a complement to the first we performed it only for GO terms that were significant at the gene level. To perform this second test we first assigned GO terms to all 50kb regions in the genome based on the GO terms assigned to overlapping genes. This analysis included both sweep regions and non sweep regions. A p-value based on Fisher's exact test was then calculated by counting the number of sweep regions (a subset of all 50kb regions) with a given term and comparing this to the background count across all regions.

Estimating the timing of selection at the peroxinectin locus

To investigate the timing of the selective sweep on the peroxinectin locus we used the R package starTMRCA (commit cf9f021 from github)(Smith et al. 2018) which estimates the time to the common ancestor (TMRCA) of haplotypes bearing a beneficial allele based on the length distribution of ancestral haplotypes and the accumulation of mutations since divergence. Since we did not know the beneficial allele, we instead identified alleles likely to be in complete linkage with the beneficial allele to serve as its proxy. We did this by choosing sites for which the derived allele was nearly fixed (on all but 3 haplotypes) in the inshore population and completely absent offshore. There were 84 such SNPs within the sweep locus, of which 75 were found within the gene s0150.g24 that overlapped with the strongest statistical indicators of selection (fig 4A). Of these 75 sites we chose 3 spanning the length of the gene (at positions 278594, 281245, 282923)

We then used VCFtools to export a 1Mb region centred on s0150.g24 from our phased vcf. For each of the 3 SNPs chosen as proxies for the beneficial allele we then used the R package REHH(Gautier and Vitalis 2012) to generate a furcation plot, and phytools(Revell 2012) combined with ggtree (Yu et al. 2017) to plot a midpoint rooted neighbour joining phylogenetic tree of the core haplotypes (central 200 sites). These visualisations all produced qualitatively similar results, all showing a clear distinction between selected and background haplotypes in the tree and strong extended haplotype homozygosity in the furcation plot.

We then ran starTMRCA separately for each of the 3 chosen SNPs using the 1Mb phased vcf as input. Other parameters were as follows; mutation rate of $1.2e^{-8}$ per base per generation, a recombination rate of $3.2e^{-8}$ per base per generation, chain length of 10000, proposal standard deviation of 20, initial value of TMRCA drawn from a uniform distribution from 0-10000

generations. Convergence was checked by running 10 independent chains and calculating the Gelman diagnostic using the coda package in R. For each SNP we recorded the median value of the posterior estimates of the TMRCA after discarding the first half as burn-in. Our final estimate for the time of selection on the locus is reported as the range of estimated values across these three SNPs.

The mutation rate used for starTMRCA analyses is the same as used for fastsimcoal2 and SMC++. The recombination rate was estimated based on a linkage map for *Acropora millepora (Wang et al. 2009; Dixon et al. 2015)* which had a length of 1358 centimorgans. The rate used was then calculated by assuming a constant recombination rate and genome size of 430Mb for *A. millepora*.

Estimating allele age with GEVA

To estimate the time of origin for derived alleles in the peroxinectin locus we used Genealogical Estimation of Variant Age (GEVA)(Albers and McVean 2020). As GEVA requires polarisation of ancestral and derived alleles we performed this task first, using est-sfs(Keightley and Jackson 2018). Inputs to est-sfs were generated by performing a whole genome alignment of the A. digitifera genome to the genomes of two related species, *Acropora millepora* (GCF_013753865.1), and *Acropora tenuis* (http://aten.reefgenomics.org/) using progressive cactus v2.0.5(Armstrong et al. 2020). We then updated our phased vcf to encode the ancestral allele as the reference allele and used this vcf as input to GEVA. GEVA was run assuming an effective population size of 3e4, and used the same mutation rate used throughout ($1.2e^{-8}$ per base per generation), and the same recombination rate ($3.2e^{-8}$ per base per generation) as used for starTMRCA. As GEVA uses a single value of the effective population size (Ne) as a scaling parameter its value cannot properly reflect recent expansions and bottlenecks in demographic history (figure 2 main text). We choose to use the average value across populations estimated by SMC++ between the period 5kya and 200kya as this time period captures the recent bottleneck as well as long-term stable value seen in figure 2. We chose not to include very recent estimates of Ne from SMC++ because these could lead to an inflated value that was not representative of the majority of the time period captured by the phylogenetic tree of haplotypes.

Supplementary Figures

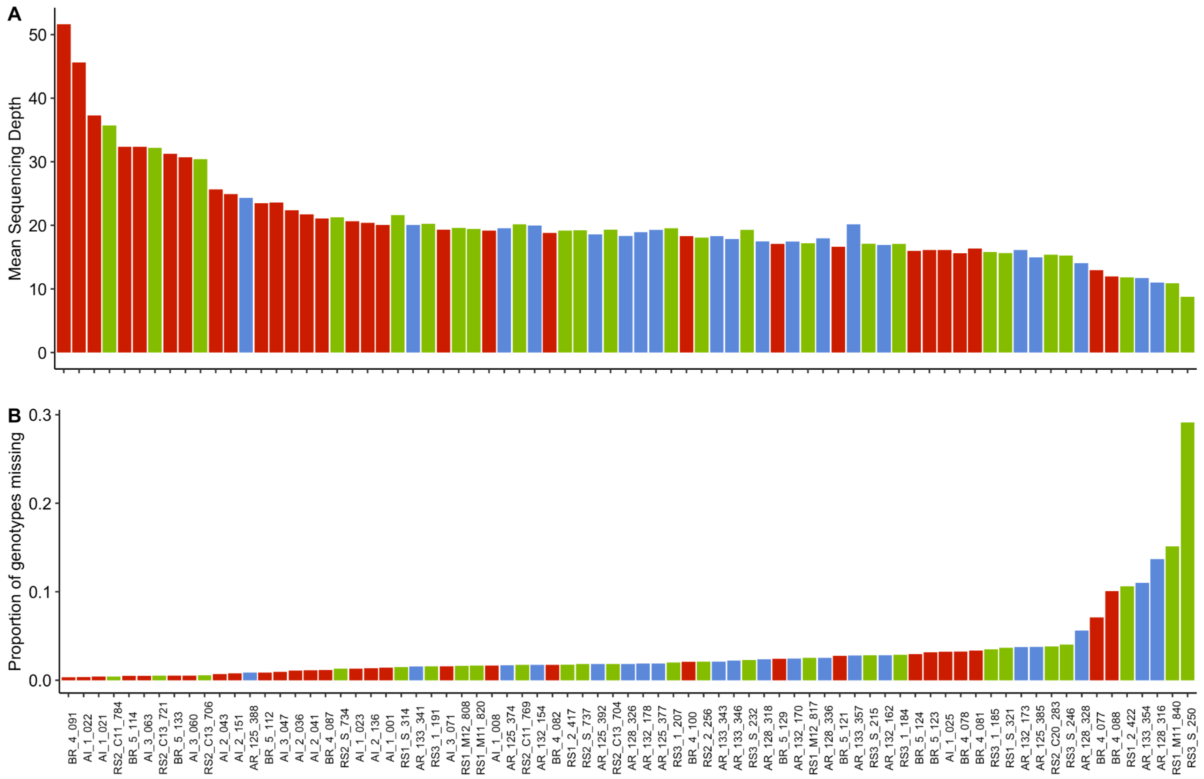

Supplementary figure S1. Mapping depth (A) and genotype missingness (B) of all samples. The colour of dots represents the sample origin population, inshore (red), north offshore (blue), and south offshore (green).

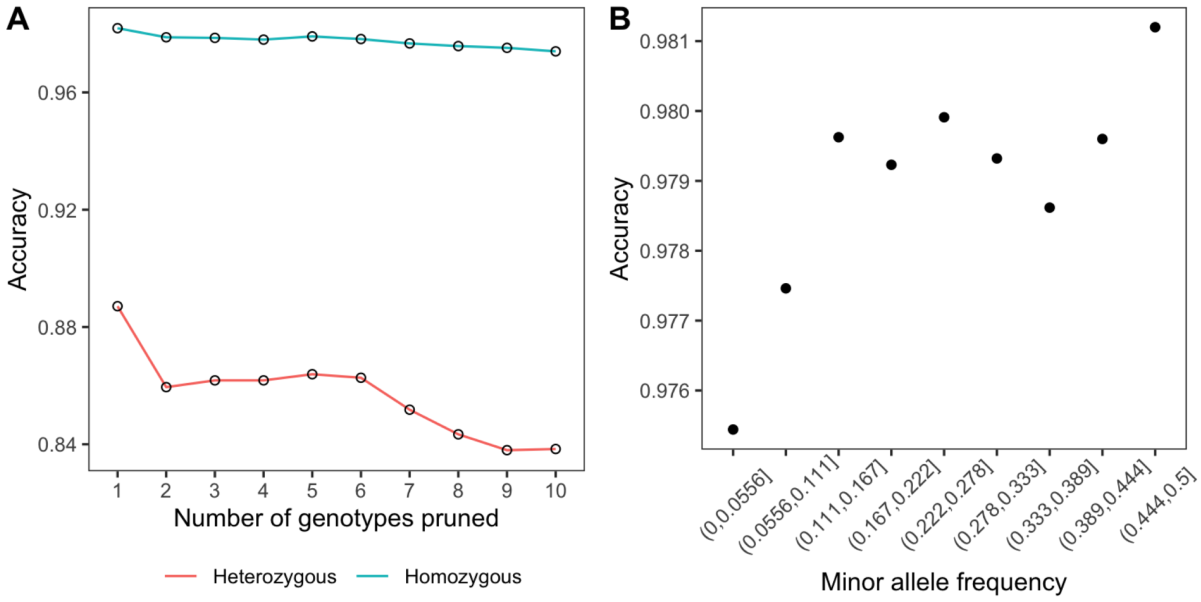

Supplementary figure S2. A. The estimated imputation accuracy at homozygous and

heterozygous sites as a function of the number of missing genotypes. B. Estimated imputation accuracy as a function of minor allele frequency

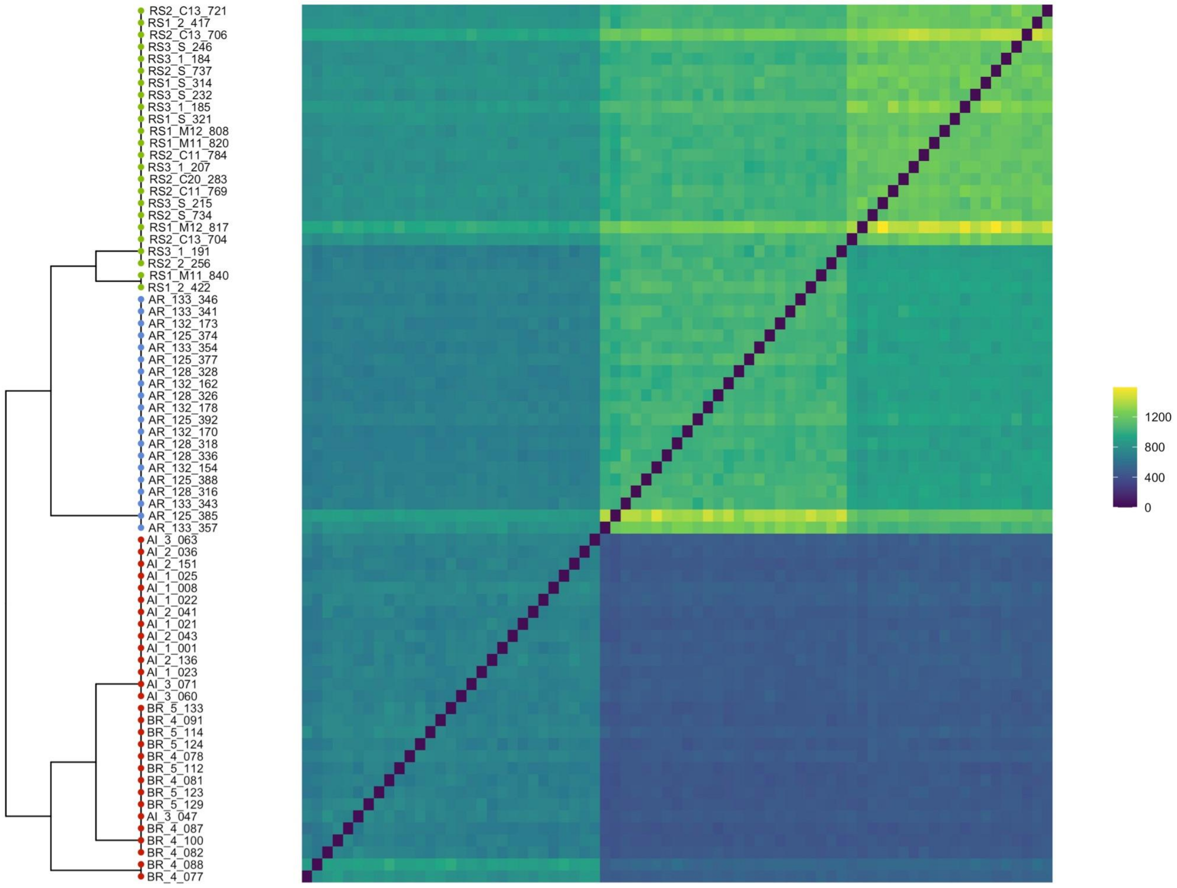

Supplementary figure S3. Sample tree and coancestry heatmap inferred by fineStructure. The tree shows nodes with greater than 99% bootstrap support and samples are coloured according to the location using the standard scheme (fig 1 in the main text). Heatmap shows coancestry between a donor sample (column) and recipient sample (row). Black line along the diagonal shows where column and row samples are the same

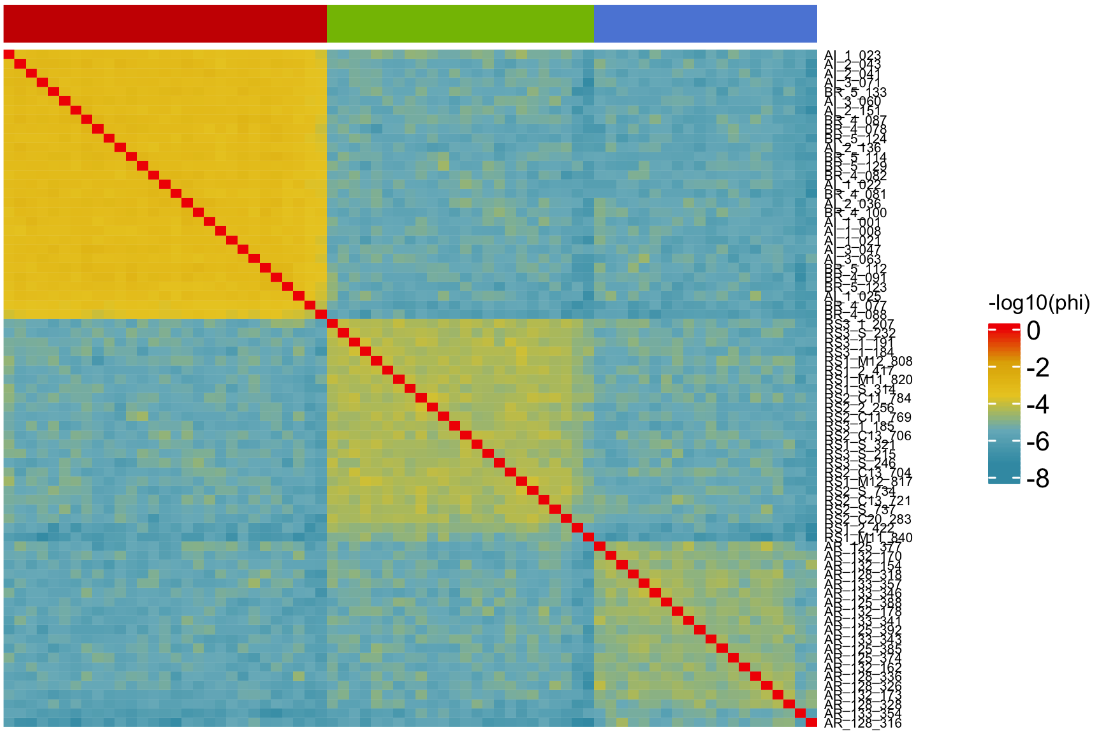

Supplementary figure S4. Clustered heatmap based on pairwise relatedness (phi) inferred by the relative proportion of IBD (identical by descent) segments. Rows and columns both represent samples and use the same clustering. Sample labels are shown for rows and column colours indicate their location using our standard colour scheme (fig 1 main text).

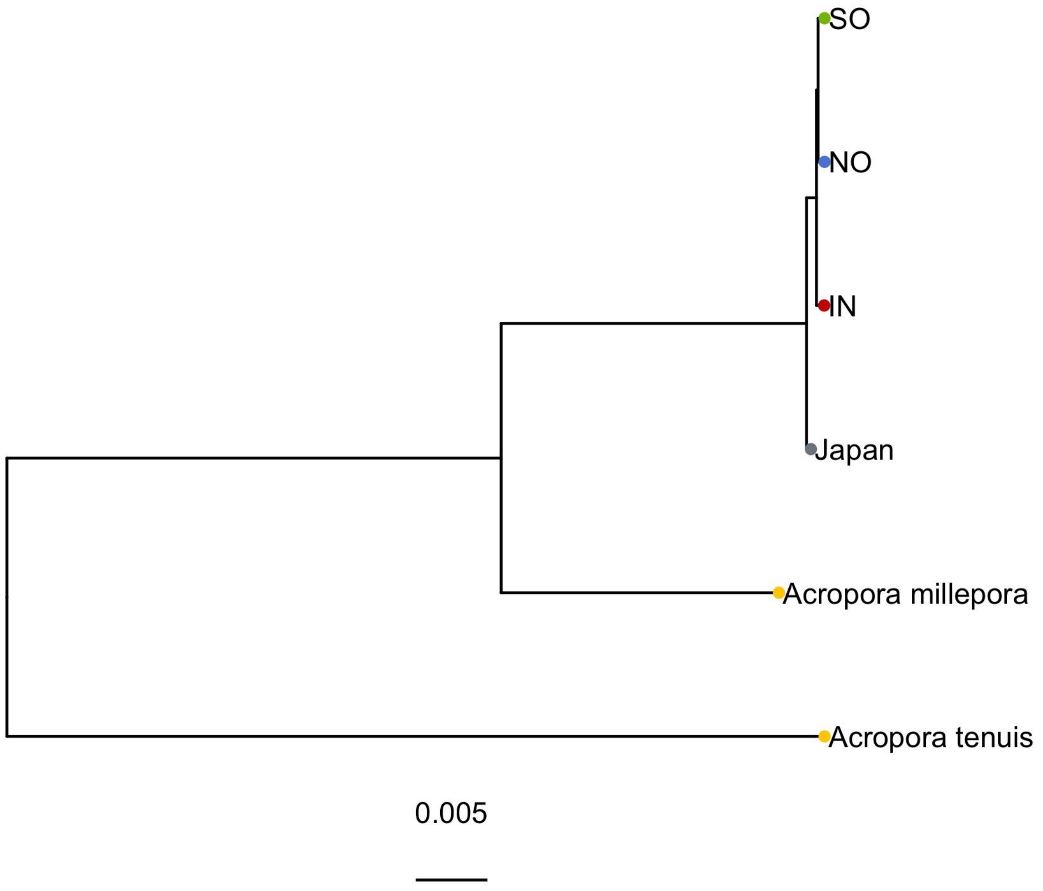

Supplementary figure S5. Phylogenetic tree showing relationships between *A. digitifera* populations from the Kimberley, Western Australia and Ryukyu Archipelago Japan, as well as outgroup species A. tenuis and A. millepora. Samples are coloured according to location using our standard colour scheme (fig1 main text) with the addition of grey to indicate samples from Japan and yellow to indicate whole genome sequences. Branch lengths incorporate both fixed substitutions and shifts in allele frequency but have been rescaled to represent substitutions per site. All clades have 100% bootstrap support based on 1000 ultrafast bootstrap replicates.

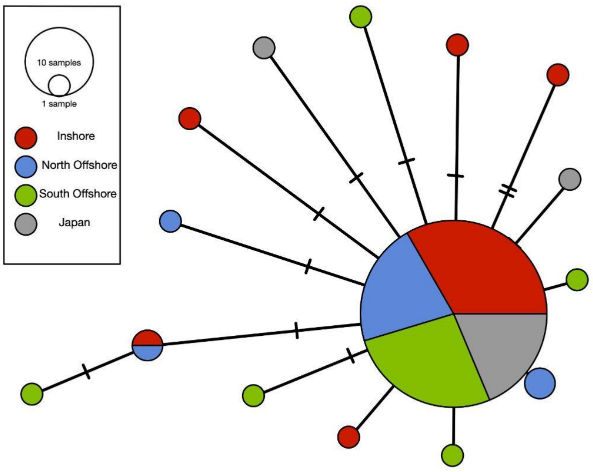

Supplementary figure S6. Haplotype network based on mitochondrial genomes of *A. digitifera* samples from the Kimberley region, Western Australia, and the Ryukyu Archipelago, Japan. Cross bars on edges indicate the number of mutations separating haplotypes while the size of nodes indicates the number of samples with the same haplotype.

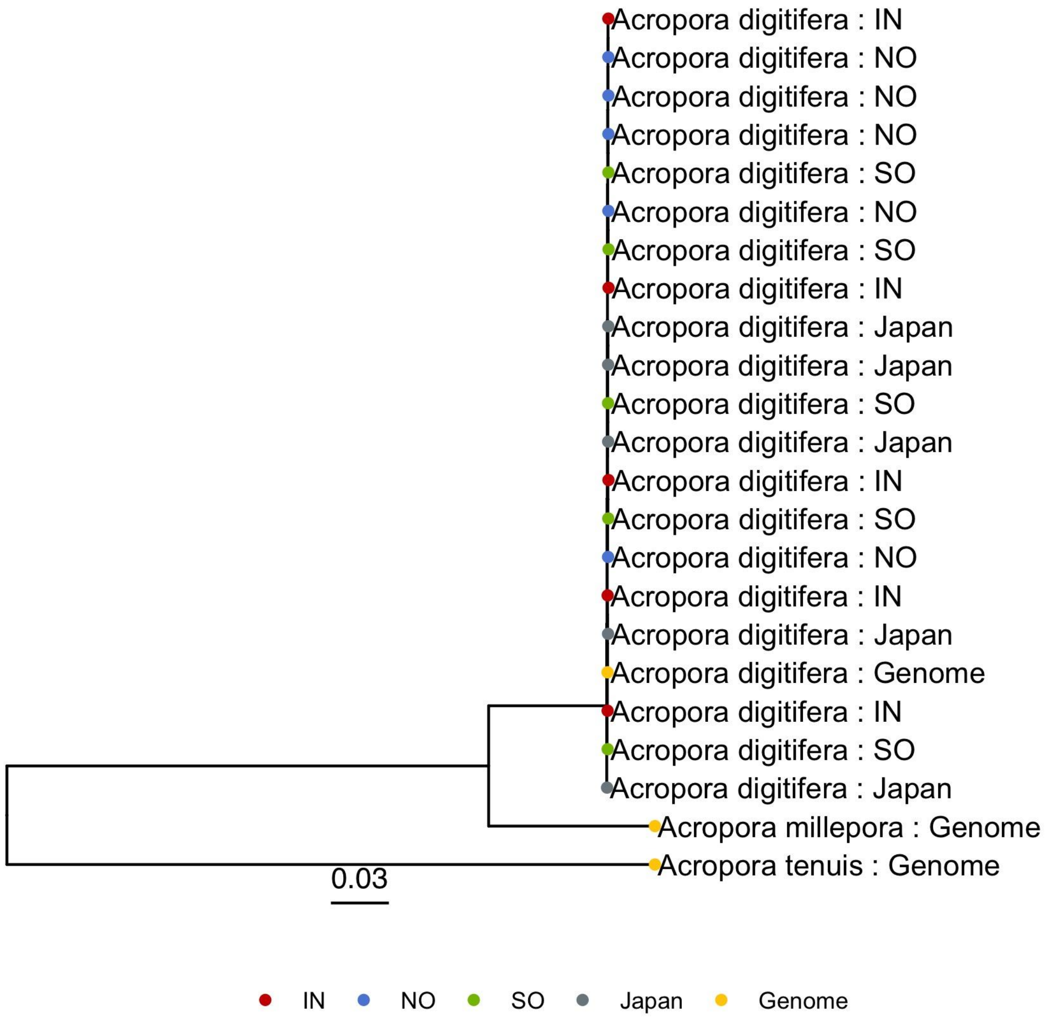

Supplementary figure S7. Maximum likelihood tree inferred from consensus UCE and Exon sequences. Sequences include those obtained from published reference genomes for *Acropora millepora* (GCF_004143615.1; (Ying et al. 2019)), *Acropora digitifera* (GCA_014634065.1, (Shinzato et al. 2020)), and *Acropora tenuis* (http://reefgenomics.org/aten/; (Cooke et al. 2020)) as well as representative population genomic samples from our study (NO, SO, IN) and from Japan (NCBI Bioproject PRJDB4188; (Shinzato et al. 2015)). Japanese samples correspond to SRA accessions (DRR099286,DRR099287,DRR099303,DRR099291,DRR099351). Samples from our study included (IN: AI_2_151, AI_3_071, AI_2_043, AI_3_047, BR_5_129; NO: AR_128_336, AR_132_154, AR_133_343, AR_132_170, AR_125_374; SO: RS1_M12_817,RS3_1_207, RS2_C13_721, RS1_S_321, RS3_1_191).

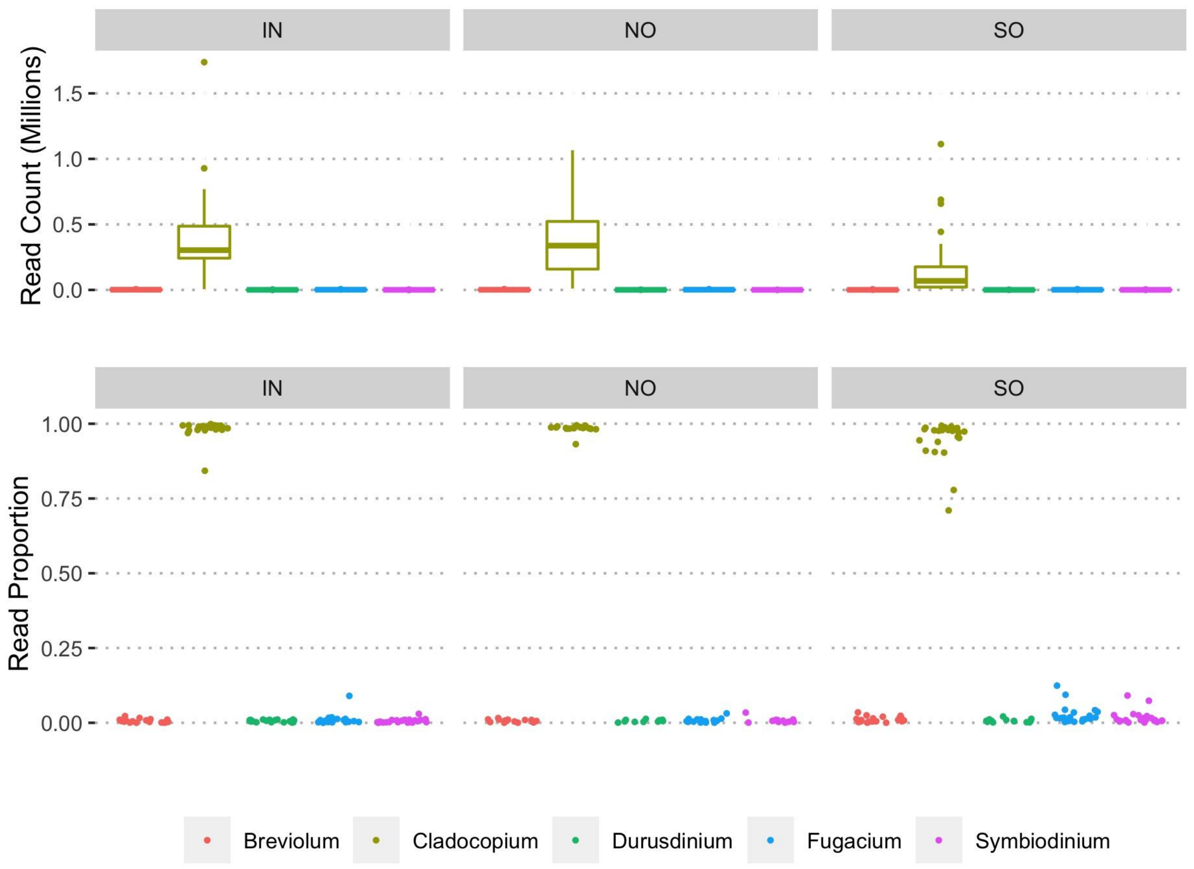

Supplementary figure S8. Summary of reads classified as Symbiodiniaceae using Kraken. Both top and bottom plots show the spread of values measured across individual samples. Read counts are shown as sample totals (top) and as proportion of the sample total (bottom) across five genera of Symbiodiniaceae. Samples are plotted separately for each location (IN: Inshore, NO: North Offshore, SO: South Offshore).

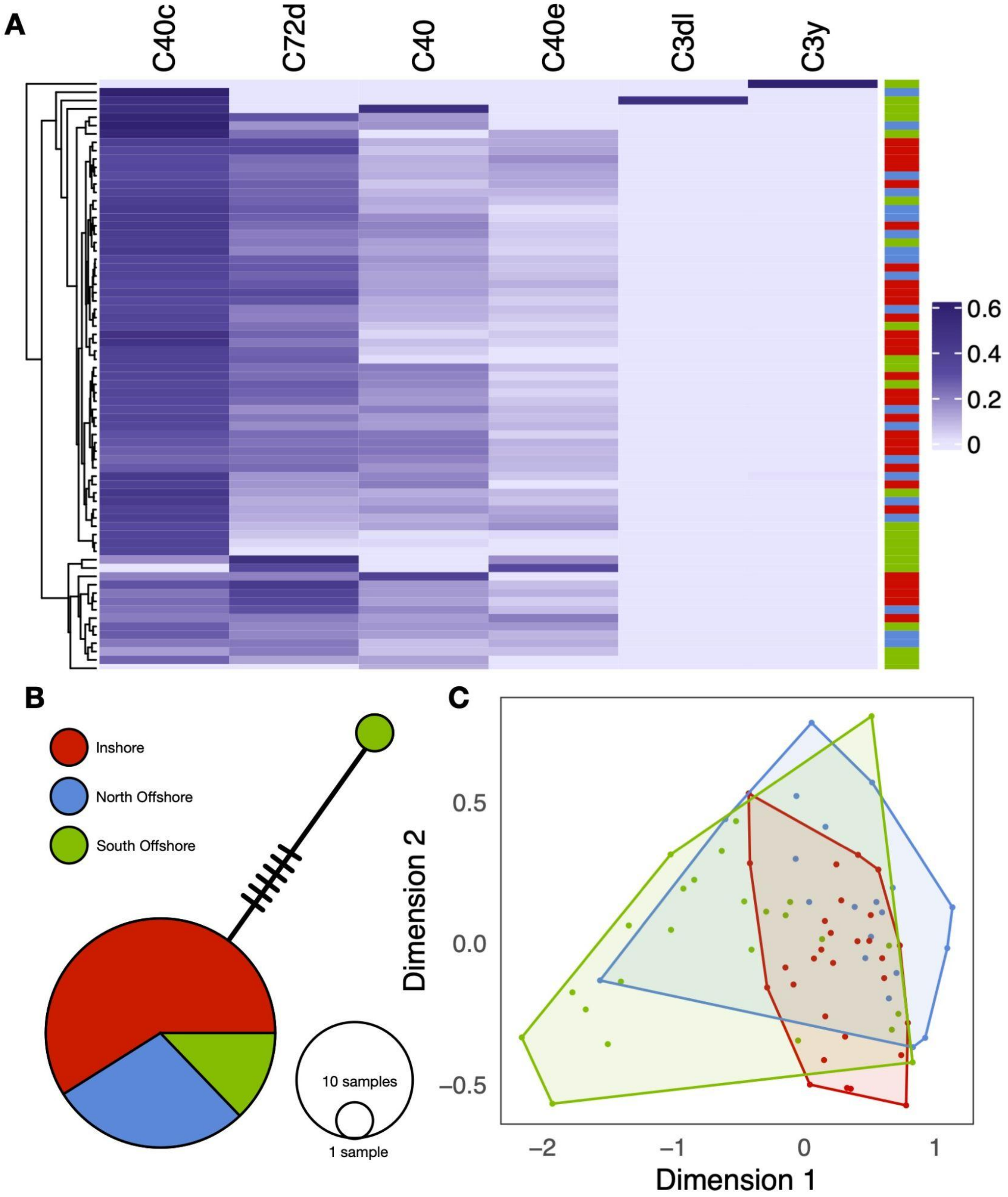

Supplementary figure S9. Diversity of sequences related to the dominant symbiont genus, *Cladocopium*. A. Heatmap of read counts mapping to symportal ITS2 reference type sequences. Rows represent coral samples, and the columns show the detected ITS2 types from read mapping. Coloured strip on the right indicates the location of origin for each sample using the colour scheme shown in B. B. Haplotype network based on mitochondrial sequences for 41 samples for which sufficient reads were available to allow consensus calling. Edge cross bars indicate the number of mutations separating haplotypes and the size of nodes indicates the number of samples with a given haplotype. C. Multidimensional

scaling (MDS) plot based on pairwise distances between samples calculated using D2S statistics. D2S statistics are calculated based on kmer counts in reads of *Cladocopium* origin (that map to the *Cladocopium goreaui* genome). Convex hulls enclose points from each location and are coloured according to our standard location color scheme (see B).

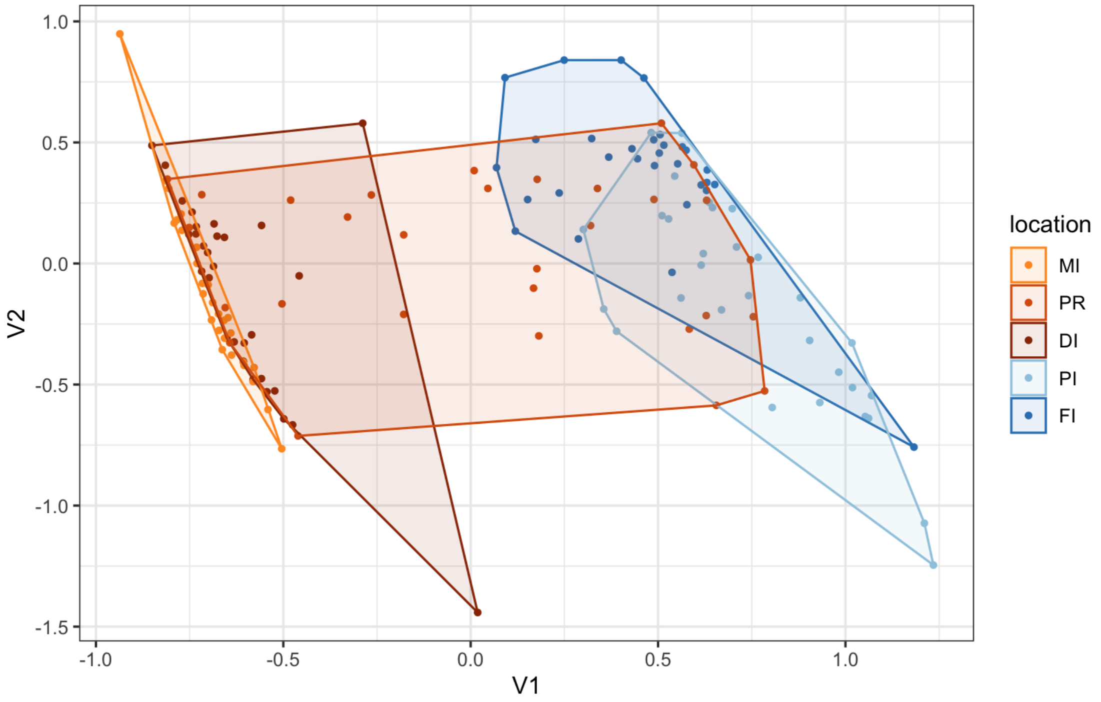

Supplementary figure S10: Multidimensional scaling (MDS) plot based on pairwise distances between Symbiodiniacea reads extracted from whole genome sequencing of *Acropora tenuis* samples from the Great Barrier Reef (Cooke et al. 2020). Distances are calculated using D2S statistics as in Figure 1E in the main text. Samples are labelled by location (n=30 per site) and colour coded according to the scheme used in (Cooke et al. 2020) Red/Brown colours represent plume locations and blue colours represent marine. Location codes are, MI (Magnetic Island), PR (Pandora Reef), DI (Dunk Island), PI (Pelorus Island), FI (Fitzroy Island). Note that partitioning of clusters shown in this plot recapitulates patterns shown in the mitochondrial haplotype network presented in Figure 2C from (Cooke et al. 2020)

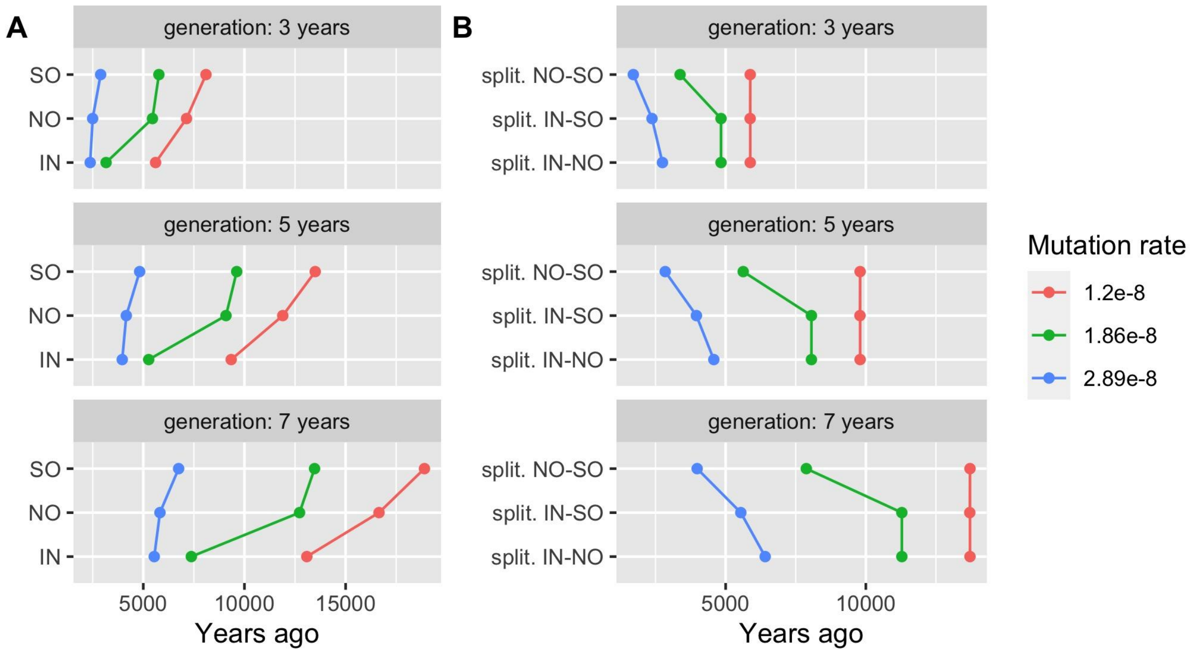

Supplementary figure S11. Variation in the estimated timing of key demographic events under different mutation rates (as mutations per base per generation) and generation times. All estimates were obtained using SMC++. A. the bottleneck time of inshore (IN), north offshore (NO), and south offshore (SO) *A. digitifera* populations. B. the split time between each pair of populations.

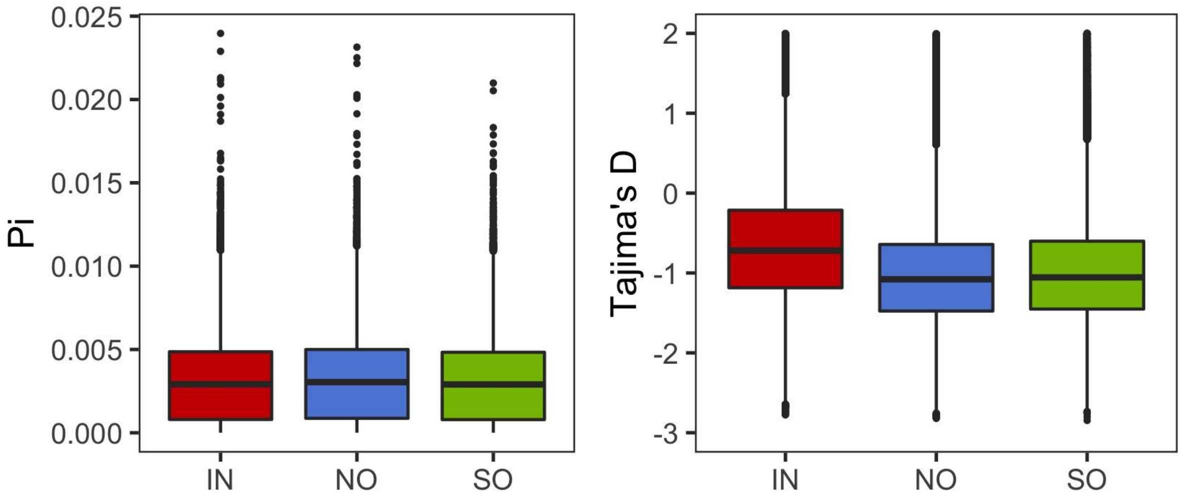

Supplementary figure S12. Boxplots showing the genome-wide distribution of nucleotide diversity (Pi; left) and of Tajima's D (right). Both plots show results for each of the three populations separately and use our standard color scheme to denote location (fig 1 main text).

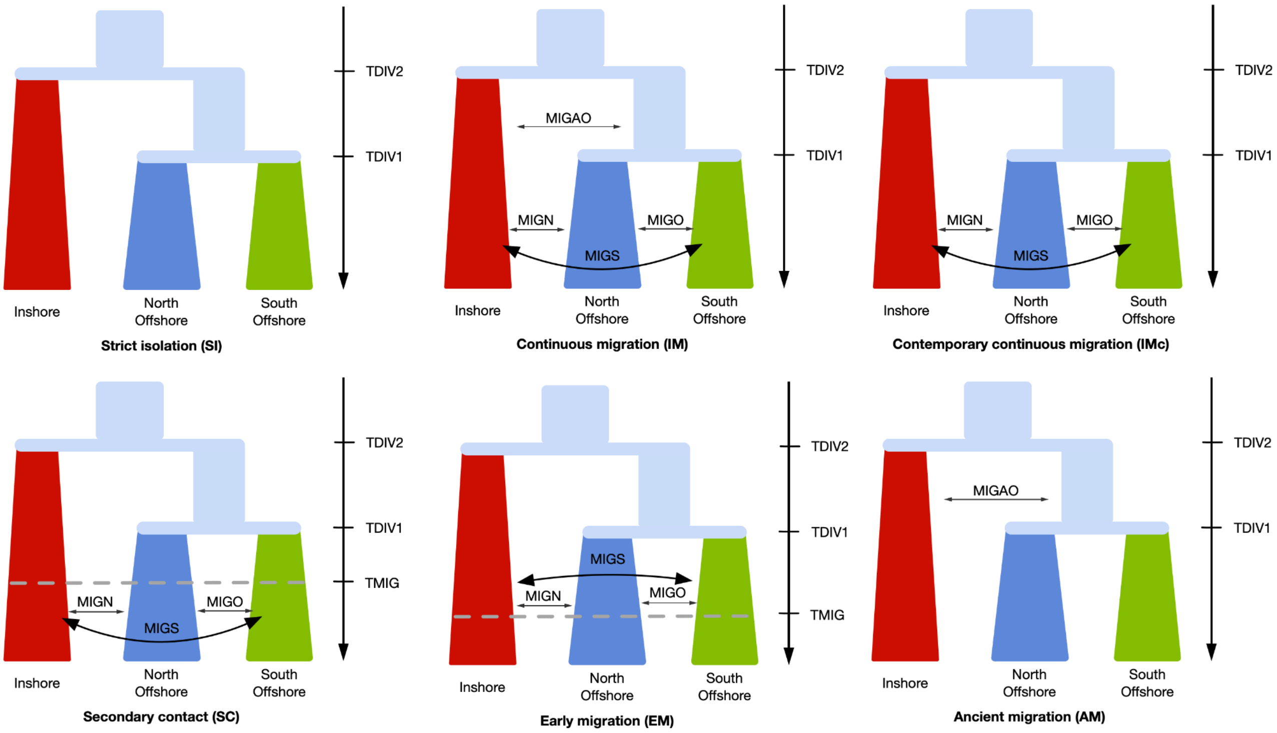

Supplementary figure S13. Schematic diagram of six alternative demographic models used in fastsimcoal2. Time is shown from most ancient (top) to present day (bottom). All models were allowed to have an exponential growth rate. The parameters TDIV1 and TDIV2 represent the time of offshore-offshore divergence and inshore-offshore divergence, respectively. Moving forward in time, TMIG represents the time at which migration starts. In models with TMIG there is no migration prior to TMIG. In models without TMIG the migration parameters persist only during one of the time intervals delineated by TDIV1 and TDIV2.

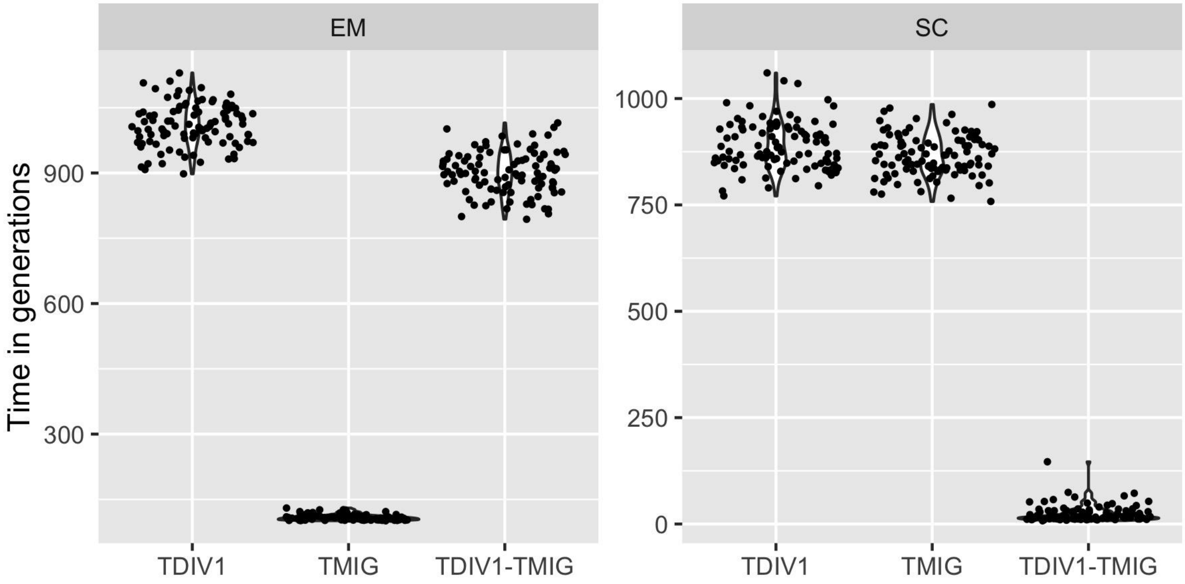

Supplementary figure S14. Estimates from fastsimcoal2 for the time of offshore-offshore divergence (TDIV1) and the migration start time (TMIG) in models EM and SC (see figure S12). Points are randomly jittered on the x-axis to avoid overplotting and show the distribution over 100 independent fastsimcoal2 runs.

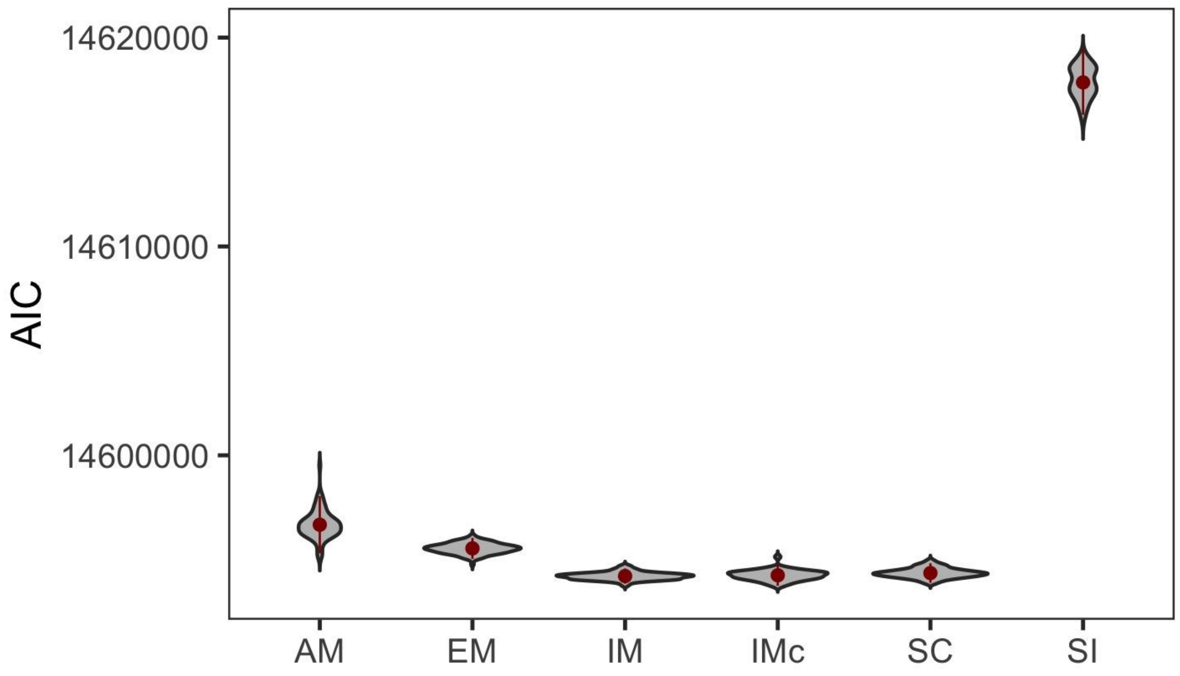

Supplementary figure S15. Distributions of AIC values from 100 independent fastsimcoal2 runs for each model described in supplementary figure S12.

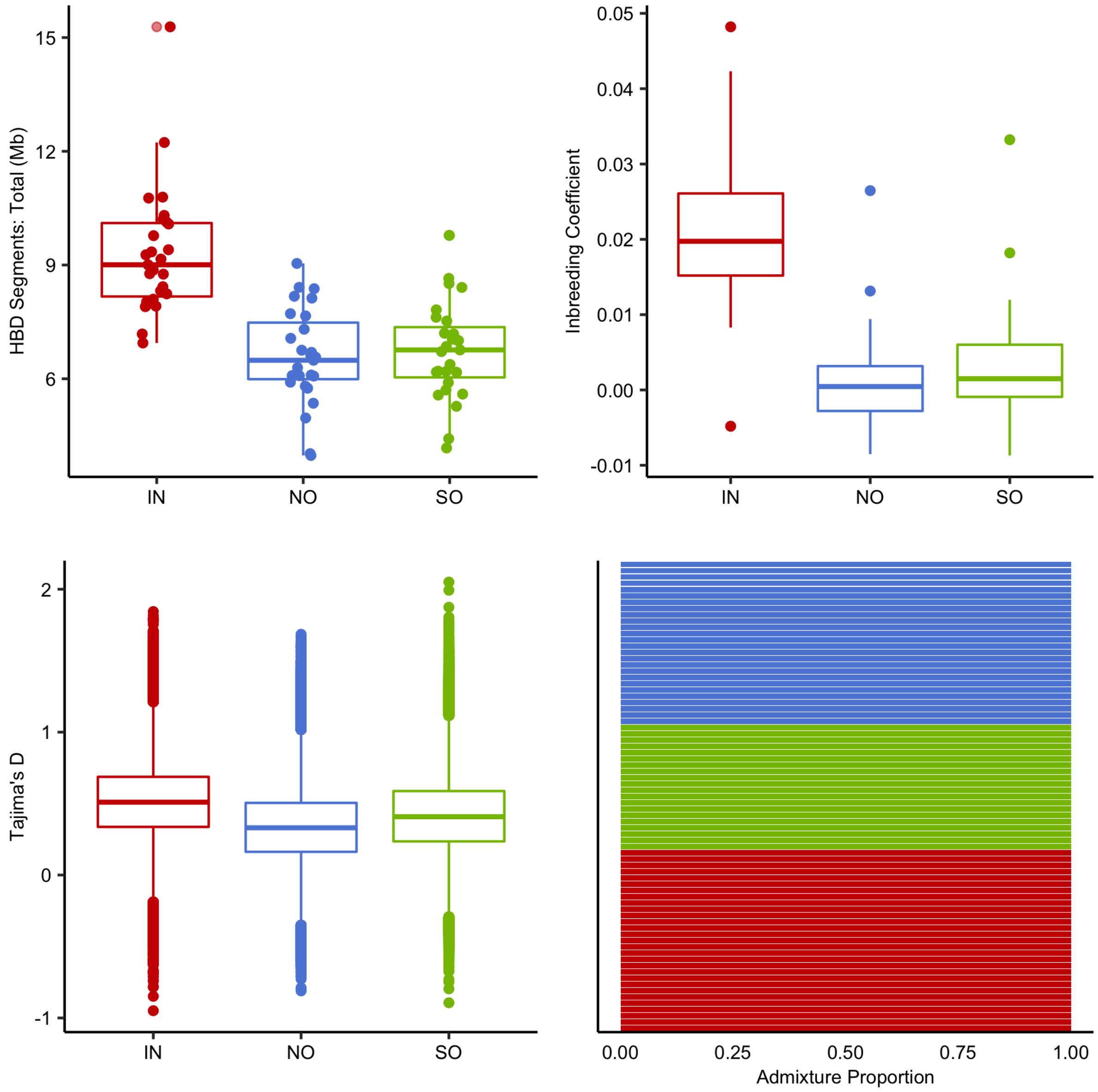

Supplementary figure S16. Population genetic statistics and Admixture proportions calculated based on simulated data under the best fitting model from fastsimcoal2 (model IMc in supplementary table S8). Sample locations are named and coloured according to our standard scheme (main text fig 1). Boxplots show the distribution of values from 50 simulation runs with fastsimcoal2 based on independent draws across the error range of model parameters. A single representative Admixture plot is shown as all simulations produced near-identical plots.

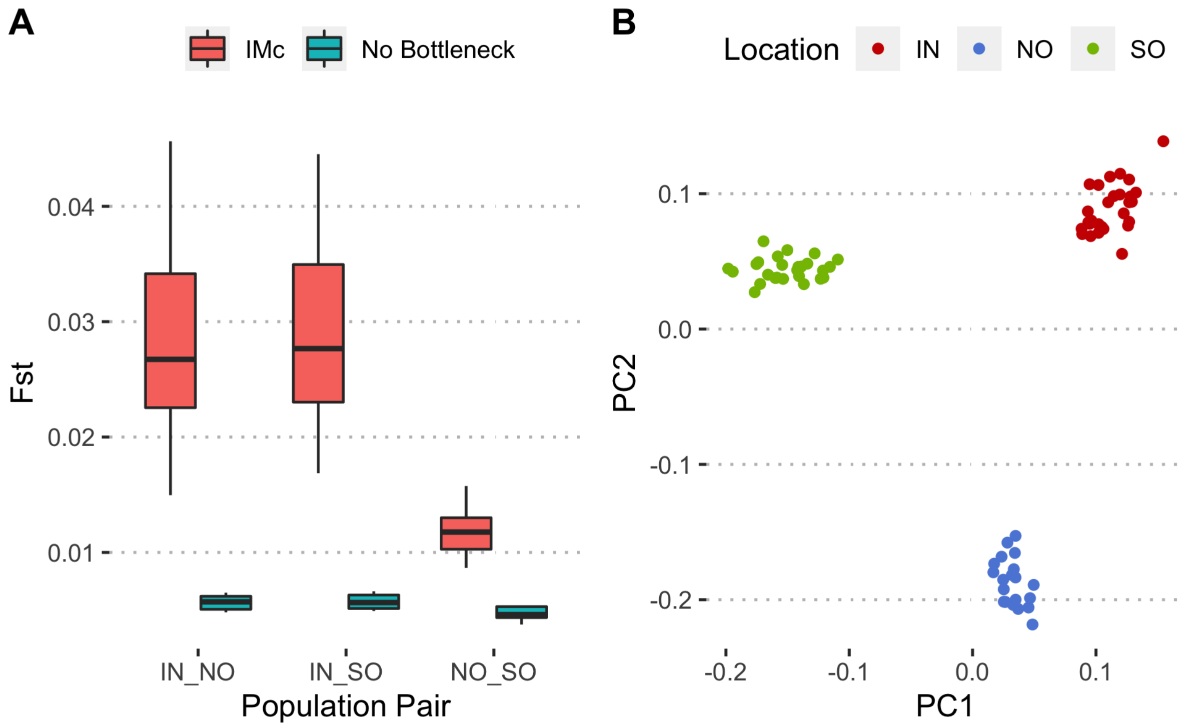

Supplementary figure S17. Influence of the bottleneck on population structure and divergence. **A.** Boxplots of pairwise Fst (Hudson) for the full IMc model compared with a model with constant population size (No Bottleneck). Spread of values is from 10 independent simulations for each model. **B.** PCA showing population structure under the No Bottleneck model. PCA shows results for one simulation. Other replicates displayed qualitatively similar results.

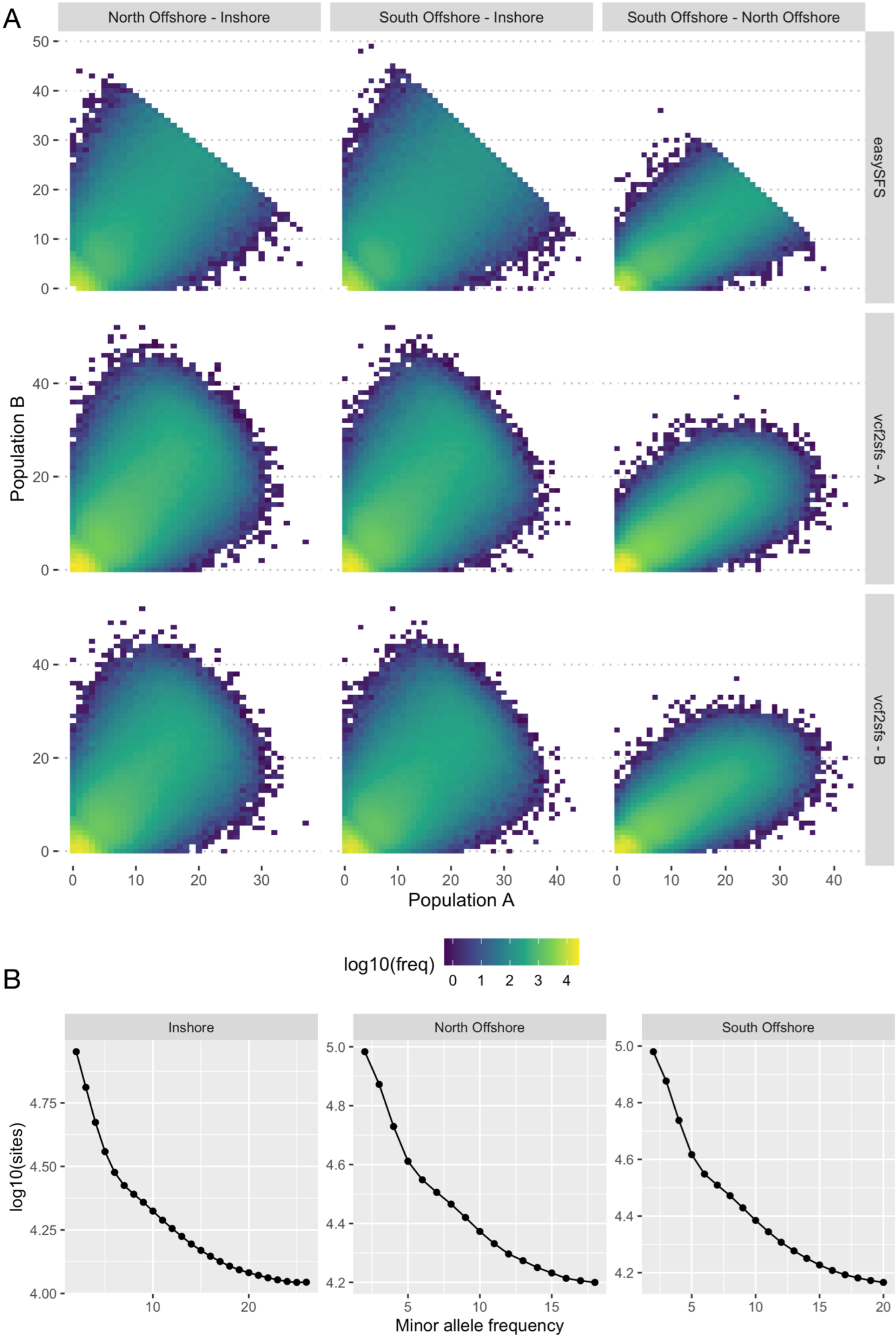

A
North Offshore - Inshore
South Offshore - Inshore
South Offshore - North Offshore
easySFS
vcf2sfs - A
vcf2sfs - B
Population B
Population A
log10(freq)
B
Inshore
North Offshore
South Offshore
log10(sites)
Minor allele frequency

Supplementary figure S18. A. Two dimensional folded site frequency spectra for all pairs of Western Australian A. digitifera populations. Labels (top) are written as population A - population B, where the allele frequency in A is shown on the horizontal axis and the allele frequency in B is shown on the vertical axis. Spectra resulting from three different vcf to SFS conversion methods and input datasets are shown. easySFS is the SFS used throughout the manuscript. vcf2sfs - A is generated based on phased input data using scripts vcf2sfs, foldSFS and SFSTools.R (as described in methods). vcf2sfs - B was generated using the same pipeline as vcf2sfs - A but after the removal of sites within 500kb of putative sweep regions. Monomorphic sites (lower left corner) have been removed to improve the colour scale. Note that highly diverged barrier loci should be visible as high frequency bands along horizontal/vertical axes under a scenario of ancient divergence with secondary contact and heterogenous gene flow. **B.** 1D projections of the SFS generated using easySFS. Note that a broad "shoulder" is visible in all plots between MAF 5-10 and this is also visible as a dark diagonal band in the lower left corner of all plots in A.

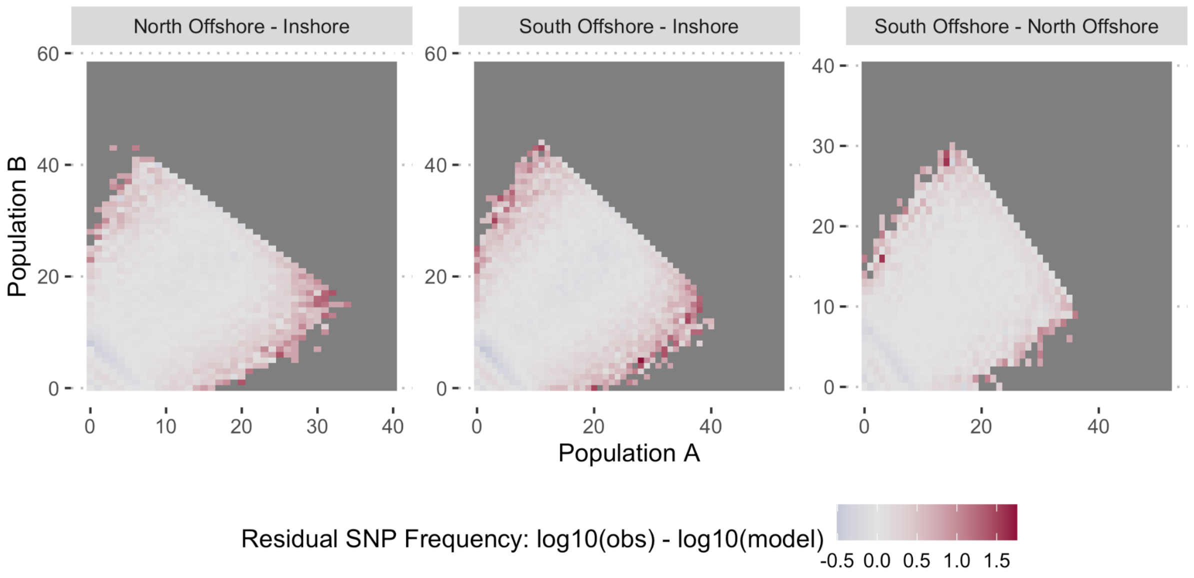

Supplementary figure S19. Residuals plots showing difference between observed SFS and modeled values based on the best fit to the IMc model. Labels (top) are written as population A - population B, where the allele frequency in A is shown on the horizontal axis and the allele frequency in B is shown on the vertical axis. White regions show areas of good fit. The lower left of all plots shows alternating grey and red bands reflecting deviations between modelled and observed spectra in this region. Note that the edges of the SFS are supported by the least data resulting in more noise (red; observed values too high or grey; observed values too low resulting in missing data).

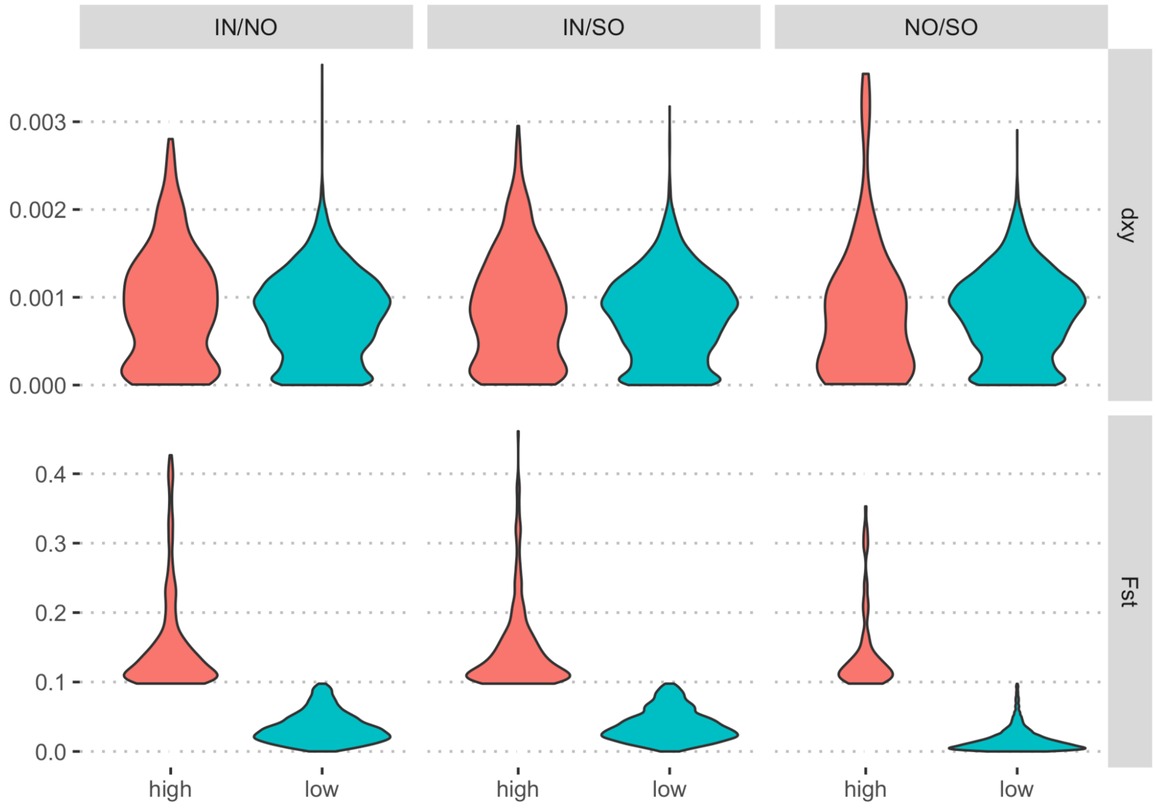

Supplementary figure S20. Comparison of the distribution of Fst and dxy calculated in 50kb sliding windows across the genome. All windows are classified as having either "high" Fst (top 5%) or "low" (remainder). Violin plots show the distribution of Fst and dxy for all windows separated into these categories, and for all pairs of populations. A Wilcoxon-Rank test performed using the wilcox.test function in R for pairs IN/NO and IN/SO was significant ($p=0.008$; $p=0.02$ respectively), indicating an increase in dxy in regions of elevated Fst, the magnitude of change was small (11.8%, 10% of the mean respectively) for these pairs.

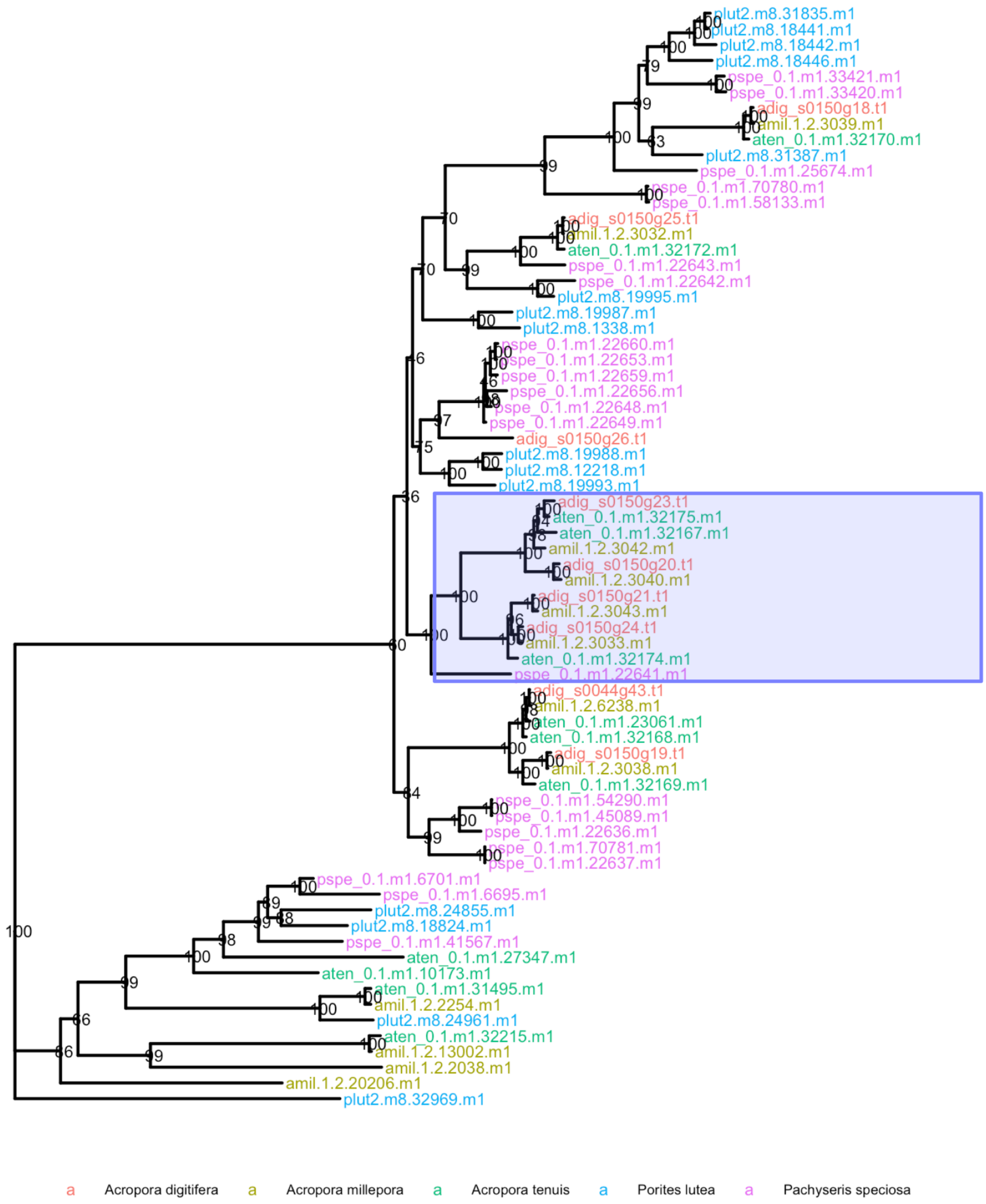

Supplementary figure S21. Phylogenetic relationships among haem peroxidases in representative coral genomes. Species chosen include three representatives of the genus Acropora and two outgroups, *Porites lutea* and *Pachyseris speciosa*. Highlighted clade includes four genes from the peroxinectin locus in A. digitifera that was examined in detail in the main text. All genes within the highlighted clade form clusters (closely spaced within the genome) in their respective species. The phylogeny shown is a subtree of the full phylogeny of haem peroxidases that includes all 8 members of the co-located peroxinectin cluster in *A. digitifera* as well as an outgroup used to root the tree. Nodes show bootstrap values based on 1000 ultrafast bootstrap replicates in IQ-Tree.

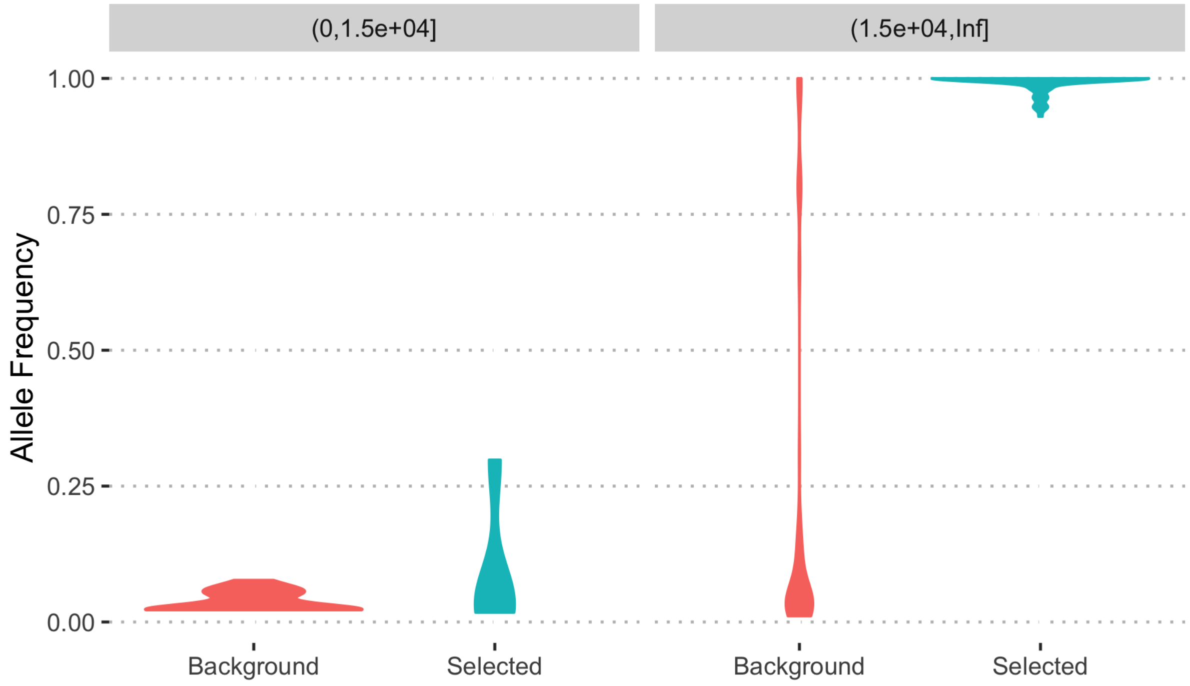

Supplementary figure S22. Relationship between frequency and age for derived alleles at 178 SNPs within the gene s0150.g24. All SNPs for which GEVA was able to calculate an age are included except those that were monomorphic compared with the reference. Distributions of allele frequencies are shown using violin plots and split by age class (0-15Kya : left) and (>15Kya : right). For each allele its frequency in background haplotypes (red) is calculated separately from in selected haplotypes (blue).

References:

Adam AAS, Thomas L, Underwood J, Gilmour J, Richards ZT. 2022. Population connectivity and genetic offset in the spawning coral Acropora digitifera in Western Australia. *Mol. Ecol.* [Internet]. Available from: https://onlinelibrary.wiley.com/doi/abs/10.1111/mec.16498

Albers PK, McVean G. 2020. Dating genomic variants and shared ancestry in population-scale sequencing data. *PLoS Biol.* 18:e3000586.

Alexa A, Rahnenführer J, Lengauer T. 2006. Improved scoring of functional groups from gene expression data by decorrelating GO graph structure. *Bioinformatics* 22:1600–1607.

Alonge M, Soyk S, Ramakrishnan S, Wang X, Goodwin S, Sedlazeck FJ, Lippman ZB, Schatz MC. 2019. RaGOO: fast and accurate reference-guided scaffolding of draft genomes. *Genome Biol.* 20:224.

Armstrong J, Hickey G, Diekhans M, Fiddes IT, Novak AM, Deran A, Fang Q, Xie D, Feng

S, Stiller J, et al. 2020. Progressive Cactus is a multiple-genome aligner for the thousand-genome era. *Nature* 587:246–251.

Bairoch A, Apweiler R. 2000. The SWISS-PROT protein sequence database and its supplement TrEMBL in 2000. *Nucleic Acids Res.* 28:45–48.

Cooke I, Ying H, Forêt S, Bongaerts P, Strugnell JM, Simakov O, Zhang J, Field MA, Rodriguez-Lanetty M, Bell SC, et al. 2020. Genomic signatures in the coral holobiont reveal host adaptations driven by Holocene climate change and reef specific symbionts. *Sci Adv* [Internet] 6. Available from: http://dx.doi.org/10.1126/sciadv.abc6318

Danecek P, Auton A, Abecasis G, Albers CA, Banks E, DePristo MA, Handsaker RE, Lunter G, Marth GT, Sherry ST, et al. 2011. The variant call format and VCFtools. *Bioinformatics* 27:2156–2158.

Danecek P, Bonfield JK, Liddle J, Marshall J, Ohan V, Pollard MO, Whitwham A, Keane T, McCarthy SA, Davies RM, et al. 2021. Twelve years of SAMtools and BCFtools. *Gigascience* [Internet] 10. Available from: http://dx.doi.org/10.1093/gigascience/giab008

Delaneau O, Marchini J, Zagury J-F. 2011. A linear complexity phasing method for thousands of genomes. *Nat. Methods* 9:179–181.

Dixon GB, Davies SW, Aglyamova GA, Meyer E, Bay LK, Matz MV. 2015. CORAL REEFS. Genomic determinants of coral heat tolerance across latitudes. *Science* 348:1460–1462.

Excoffier L, Dupanloup I, Huerta-Sánchez E, Sousa VC, Foll M. 2013. Robust demographic inference from genomic and SNP data. *PLoS Genet.* 9:e1003905.

Excoffier L, Marchi N, Marques DA, Matthey-Doret R, Gouy A, Sousa VC. 2021. fastsimcoal2: demographic inference under complex evolutionary scenarios. *Bioinformatics* 37:4882–4885.

Fuller ZL, Mocellin VJL, Morris LA, Cantin N, Shepherd J, Sarre L, Peng J, Liao Y, Pickrell J, Andolfatto P, et al. 2020. Population genetics of the coral Acropora millepora: Toward genomic prediction of bleaching. *Science* [Internet] 369. Available from: http://dx.doi.org/10.1126/science.aba4674

Garrison E, Marth G. 2012. Haplotype-based variant detection from short-read sequencing. *arXiv [q-bio.GN]* [Internet]. Available from: http://arxiv.org/abs/1207.3907

Gautier M, Vitalis R. 2012. rehh: an R package to detect footprints of selection in genome-wide SNP data from haplotype structure. *Bioinformatics* 28:1176–1177.

Jones P, Binns D, Chang H-Y, Fraser M, Li W, McAnulla C, McWilliam H, Maslen J, Mitchell A, Nuka G, et al. 2014. InterProScan 5: genome-scale protein function classification. *Bioinformatics* 30:1236–1240.

Keightley PD, Jackson BC. 2018. Inferring the Probability of the Derived vs. the Ancestral Allelic State at a Polymorphic Site. *Genetics* 209:897–906.

Köster J, Rahmann S. 2012. Snakemake—a scalable bioinformatics workflow engine. *Bioinformatics* 28:2520–2522.

Kuhn RM, Haussler D, Kent WJ. 2013. The UCSC genome browser and associated tools. *Brief. Bioinform.* 14:144–161.

Li H. 2014. Toward better understanding of artifacts in variant calling from high-coverage samples. *Bioinformatics* 30:2843–2851.

Pertea G, Pertea M. 2020. GFF Utilities: GffRead and GffCompare. *F1000Res.* [Internet] 9. Available from: http://dx.doi.org/10.12688/f1000research.23297.2

Pfeifer B, Wittelsbürger U, Ramos-Onsins SE, Lercher MJ. 2014. PopGenome: An Efficient Swiss Army Knife for Population Genomic Analyses in R. *Mol. Biol. Evol.* 31:1929–1936.

Revell LJ. 2012. phytools: an R package for phylogenetic comparative biology (and other things). *Methods Ecol Evol* 3:217–223.

Shinzato C, Khalturin K, Inoue J, Zayasu Y, Kanda M, Kawamitsu M, Yoshioka Y, Yamashita H, Suzuki G, Satoh N. 2020. Eighteen Coral Genomes Reveal the Evolutionary Origin of Acropora Strategies to Accommodate Environmental Changes. *Mol. Biol. Evol.* 38:16–30.

Shinzato C, Mungpakdee S, Arakaki N, Satoh N. 2015. Genome-wide SNP analysis explains coral diversity and recovery in the Ryukyu Archipelago. *Sci. Rep.* 5:18211.

Sims D, Sudbery I, Ilott NE, Heger A, Ponting CP. 2014. Sequencing depth and coverage: key considerations in genomic analyses. *Nat. Rev. Genet.* 15:121–132.

Smith J, Coop G, Stephens M, Novembre J. 2018. Estimating Time to the Common Ancestor for a Beneficial Allele. *Mol. Biol. Evol.* 35:1003–1017.

Tine M, Kuhl H, Gagnaire P-A, Louro B, Desmarais E, Martins RST, Hecht J, Knaust F, Belkhir K, Klages S, et al. 2014. European sea bass genome and its variation provide insights into adaptation to euryhalinity and speciation. *Nat. Commun.* 5:5770.

Verma SS, de Andrade M, Tromp G, Kuivaniemi H, Pugh E, Namjou-Khales B, Mukherjee S, Jarvik GP, Kottyan LC, Burt A, et al. 2014. Imputation and quality control steps for combining multiple genome-wide datasets. *Front. Genet.* 5:370.

Wang S, Zhang L, Meyer E, Matz MV. 2009. Construction of a high-resolution genetic linkage map and comparative genome analysis for the reef-building coral Acropora millepora. *Genome Biol.* 10:R126.

Ying H, Hayward DC, Cooke I, Wang W, Moya A, Siemering KR, Sprungala S, Ball EE, Forêt S, Miller DJ. 2019. The Whole-Genome Sequence of the Coral Acropora millepora. *Genome Biol. Evol.* 11:1374–1379.

Yu G, Smith DK, Zhu H, Guan Y, Lam TT-Y. 2017. Ggtree : An r package for visualization and annotation of phylogenetic trees with their covariates and other associated data. *Methods Ecol. Evol.* 8:28–36.
